# Pink-beam serial femtosecond crystallography for accurate structure-factor determination at an X-ray free-electron laser

**DOI:** 10.1107/S2052252521008046

**Published:** 2021-09-23

**Authors:** Karol Nass, Camila Bacellar, Claudio Cirelli, Florian Dworkowski, Yaroslav Gevorkov, Daniel James, Philip J. M. Johnson, Demet Kekilli, Gregor Knopp, Isabelle Martiel, Dmitry Ozerov, Alexandra Tolstikova, Laura Vera, Tobias Weinert, Oleksandr Yefanov, Jörg Standfuss, Sven Reiche, Christopher J. Milne

**Affiliations:** a Paul Scherrer Institut, Forschungstrasse 111, Villigen 5232, Switzerland; b Center for Free-Electron Laser Science, Deutsches Elektronen-Synchrotron DESY, Notkestrasse 85, Hamburg 22607, Germany

**Keywords:** pink beams, serial femtosecond crystallography, *de novo* protein structure determination, single-wavelength anomalous diffraction, XFELs, large bandwidths, data-quality indicators

## Abstract

Serial femtosecond crystallography (SFX) at X-ray free-electron lasers (XFELs) enables essentially radiation-damage-free macromolecular structure determination using microcrystals that are too small for synchrotron studies. However, SFX experiments often require large amounts of sample in order to collect highly redundant data where some of the many stochastic errors can be averaged out to determine accurate structure-factor amplitudes. In this work, the capability of the Swiss X-ray free-electron laser (SwissFEL) was used to generate large-bandwidth X-ray pulses [Δλ/λ = 2.2% full width at half-maximum (FWHM)], which were applied in SFX with the aim of improving the partiality of Bragg spots and thus decreasing sample consumption while maintaining the data quality. Sensitive data-quality indicators such as anomalous signal from native thaumatin micro-crystals and *de novo* phasing results were used to quantify the benefits of using pink X-ray pulses to obtain accurate structure-factor amplitudes. Compared with data measured using the same setup but using X-ray pulses with typical quasi-monochromatic XFEL bandwidth (Δλ/λ = 0.17% FWHM), up to fourfold reduction in the number of indexed diffraction patterns required to obtain similar data quality was achieved. This novel approach, pink-beam SFX, facilitates the yet underutilized *de novo* structure determination of challenging proteins at XFELs, thereby opening the door to more scientific breakthroughs.

## Abstract 2

Large-bandwidth X-ray free-electron laser pulses significantly reduce sample and beam-time quantities required for obtaining accurate structure-factor amplitudes in serial femtosecond crystallography.

## 1. Introduction   

Serial femtosecond crystallography (SFX) at X-ray free-electron lasers (XFELs) offers unique opportunities for structural biologists (Chapman *et al.*, 2011 ▸; Martin-Garcia *et al.*, 2016 ▸; Orville, 2020 ▸). Owing to ultra-short and extremely intense X-ray pulses with a peak brilliance which exceeds that of third-generation synchrotron sources by nine orders of magnitude, this method allows obtaining essentially damage-free X-ray diffraction data from systems prone to radiation damage such as micrometre-sized protein crystals or metalloproteins (Barty *et al.*, 2012 ▸; Lomb *et al.*, 2011 ▸; Nass, 2019 ▸). Examples include, but are not limited to, crystals grown *in vivo* (Nass, Redecke *et al.*, 2020 ▸, 2013 ▸; Sawaya *et al.*, 2014 ▸); membrane proteins crystallized in lipidic cubic phase (LCP), such as G-protein-coupled receptors (GPCRs) (Im *et al.*, 2020 ▸; Johansson *et al.*, 2019 ▸; Kang *et al.*, 2015 ▸; Lee *et al.*, 2020 ▸; Liu *et al.*, 2013 ▸; Stauch *et al.*, 2019 ▸; Zhang *et al.*, 2015 ▸); and radiation-sensitive metalloproteins, such as photosystem II (Suga *et al.*, 2015 ▸), cytochrome *c* oxidase (Hirata *et al.*, 2014 ▸) and copper nitrite reductase (Halsted *et al.*, 2019 ▸; Rose *et al.*, 2021 ▸). Additionally, it opened the possibility to observe proteins in action via time-resolved crystallography on ultra-fast time scales (Barends *et al.*, 2015 ▸; Coquelle *et al.*, 2018 ▸; Dods *et al.*, 2021 ▸; Nango *et al.*, 2016 ▸; Nass, Gorel *et al.*, 2020 ▸; Nass Kovacs *et al.*, 2019 ▸; Pande *et al.*, 2016 ▸; Skopintsev *et al.*, 2020 ▸; Sorigué *et al.*, 2021 ▸; Woodhouse *et al.*, 2020 ▸; Yun *et al.*, 2021 ▸; Nogly *et al.*, 2018 ▸).

Despite over a decade of continuous improvements in beamline instrumentation, X-ray detection performance, data-processing software, sample delivery and structure-determination methods for SFX, elucidation of unknown protein structures at XFEL sources remains severely underutilized. Several SFX studies have demonstrated that *de novo* structure determination from SFX data is possible using well characterized and strongly diffracting model systems via single-wavelength anomalous diffraction (SAD) (Barends *et al.*, 2014 ▸; Hunter *et al.*, 2016 ▸; Nakane *et al.*, 2016 ▸; Sugahara *et al.*, 2017 ▸; Yamashita *et al.*, 2017 ▸), native SAD (Batyuk *et al.*, 2016 ▸; Nakane *et al.*, 2015 ▸; Nass *et al.*, 2016 ▸), isomorphous replacement (Yamashita *et al.*, 2015 ▸), and novel phasing methods such as simultaneous multi-wavelength anomalous diffraction (MAD) with two-color X-ray pulses (Gorel *et al.*, 2017 ▸) and high-intensity MAD (Son *et al.*, 2011 ▸), but to date only one previously unknown structure of BinAB was determined at an XFEL (Colletier *et al.*, 2016 ▸).

One of the most significant reasons for this underutilization of SFX for *de novo* structure determination, in addition to the scarcity of available beam time, are various sources of experimental errors that make it challenging to obtain accurate measurements of small signals required for phasing. They arise from stochastic fluctuations of X-ray pulse properties, background scattering from media used to deliver hydrated crystals to the interaction region with X-ray pulses and difficulties in analyzing still diffraction patterns from crystals in random orientations with unknown partiality of individual reflections. As an example of efforts to overcome these limitations, partiality correction was introduced into SFX data-processing software (Ginn *et al.*, 2015 ▸, 2016 ▸; Kabsch, 2014 ▸; Sauter, 2015 ▸; White, 2014 ▸; White *et al.*, 2016 ▸); however, in practice, this procedure often fails when other experimental errors are large. Currently, the most popular approach in SFX to decrease the experimental errors and obtain full reflection intensities from still diffraction patterns is Monte Carlo averaging (Kirian *et al.*, 2011 ▸) but it requires a large number of measurements, especially for *de novo* structure determination. This translates into larger sample quantities and longer measurement times required.

Recently, an improvement in native-SAD phasing of SFX data was demonstrated by utilizing longer wavelengths that increased the strength of the anomalous signal (Nass, Cheng *et al.*, 2020 ▸). This reduced up to tenfold the number of indexed images needed for successful *de novo* structure determination. Another approach to reduce the number of indexed images, applicable not only to *de novo* phasing but also to molecular replacement strategies, is to use polychromatic (pink) X-ray pulses for SFX. Theoretically, faster convergence rates of the Monte Carlo approach can be achieved by increasing the bandwidth (BW) or divergence of the X-ray pulses (Dejoie *et al.*, 2013 ▸; White *et al.*, 2013 ▸). This is due to improvement of partiality of individual Bragg spot intensities and increase of the number of Bragg spots per pattern owing to a larger coverage of the reciprocal lattice points located between the limiting Ewald spheres. Hence, the large-BW XFEL pulses might allow obtaining a complete set of full structure-factor amplitudes with fewer number of indexed diffraction patterns than typical self-amplified spontaneous emission (SASE) BW XFEL pulses.

Initial pink-beam serial crystallography experiments at synchrotrons were performed using a spectral BW of 2.5% (Tolstikova *et al.*, 2019 ▸) and a full harmonic of the undulator spectrum with a BW of 5.7% [full width at half-maximum (FWHM)] (Martin-Garcia *et al.*, 2019 ▸; Meents *et al.*, 2017 ▸), and show a reduction in the number of images required for a set of usable structure factors. However, data collected at synchrotrons at room temperature from micro-crystals, especially metalloproteins, are particularly affected by radiation damage. With the upcoming upgrades of synchrotron sources, brighter and smaller X-ray beams resulting in approximately two orders of magnitude larger average photon flux densities are expected to be used for (pink beam) serial crystallography experiments. As an example, serial synchrotron crystallography experiments have shown that a decay of the diffracting power by half is observed at *D*_1/2_ = 150 kGy (Roedig *et al.*, 2016 ▸), and an elongation of the iron–water bond in DtpAa, indicative of Fe^III^ to Fe^II^ reduction, is observed at a dose of 33 kGy but not in the SFX structure (Ebrahim *et al.*, 2019 ▸).

Here we demonstrate that large-BW X-ray pulses with Δλ/λ = 2.2% (FWHM) from an XFEL significantly improve SFX data quality when compared with nominal SASE BW with Δλ/λ = 0.17% (FWHM). We used microcrystals of a well characterized system (thaumatin) and X-ray pulses with a photon energy of 6 keV (2.066 Å) to perform a detailed comparison of data quality between data sets measured with large and nominal SASE BW X-ray pulses from SwissFEL (Prat, Abela *et al.*, 2020 ▸). We present up to fourfold reduction of the number of images required for obtaining similar data quality with large-BW XFEL pulses as compared with SASE using the same experimental setup and similar pulse properties. As a sensitive data-quality indicator, we used the weak anomalous signal from sulfur atoms and results from an automatic *de novo* structure-determination pipeline. The improvement offered by increased spectral BW of XFEL pulses significantly reduces the data-collection time and sample quantity needed for obtaining a high-quality SFX data set, not only for *de novo* phasing applications but also for molecular replacement strategies used, for example, in time-resolved SFX.

## 2. Methods   

### 2.1. Preparation of thaumatin microcrystals and embedding in LCP   

Preparation and embedding of thaumatin microcrystals in LCP were carried out as described (Nass, Cheng *et al.*, 2020 ▸). The average size of the microcrystals was ∼20 µm in length and ∼10 µm in width.

### 2.2. Diffraction data collection at SwissFEL   

SFX data from thaumatin microcrystals were collected in May 2019 during a regular user beam time at SwissFEL (proposal No. 20182149, ‘Large bandwidth X-ray FEL pulses for reducing partiality in SFX’). Thaumatin microcrystals embedded in LCP were delivered to the interaction region with X-ray pulses using an LCP injector (Weierstall *et al.*, 2014 ▸). The injector was mounted vertically in the Prime chamber of the Alvra experimental hutch with a distance between the tip of the nozzle and the detector surface of ∼95 mm. The diameter of the capillary used to extrude the sample was 50 µm. Pressure to drive the injector was supplied by a remotely controlled high-performance liquid chromatography (HPLC) system (Shimadzu). The flow rate set on the HPLC pump ranged from 0.002 to 0.005 ml min^−1^ resulting in a sample consumption rate of 0.15 to 0.37 µl min^−1^. An estimated distance of 50 and 125 µm between the exposed areas from two consecutive X-ray exposures ensured sufficient separation to avoid radiation damage from the previous shot. To reduce the background scattering from the residual air in the chamber, the pressure was decreased to 100 mbar, filled up to 800 mbar with He gas and then reduced again to 100 mbar prior to measurements. The temperature in the hutch was kept constant at 24°C. The X-ray pulses were attenuated to achieve both stable flow of the LCP jet and to avoid saturated Bragg spot intensities on the detector. The attenuation was adjusted when switching between the large-BW and SASE modes to keep the fluence on the sample constant. Single-shot diffraction patterns were recorded at the 25 Hz repetition rate of the X-ray laser using the low-noise automatic gain switching JUNGFRAU 16M detector (Mozzanica *et al.*, 2016 ▸) and saved on disk as HDF5 files in raw format as read out directly from the detector without any modifications in runs of 10 000 consecutive shots. A Kapton foil of 75 µm thickness was installed ∼20 mm in front of the detector to separate the detector compartment filled with N_2_ gas from He gas in the main part of the chamber, and a regulation system maintained the pressure in the two compartments at equilibrium.

### 2.3. Real-time and automatic SFX data-analysis and visualization pipeline   

An automatic SFX data-analysis and visualization pipeline developed at SwissFEL was used during the experiment. The pipeline provided information about the amount and quality of collected data during the experiments in real time, which significantly reduced the workload of the data-processing team. The pipeline applied pedestal and gain corrections as well as bad pixel masking to each diffraction image ‘on the fly’ in parallel to raw data saving, and performed peak search using a modified *peakfinder8* algorithm from *Cheetah* (Barty *et al.*, 2014 ▸). Diffraction patterns containing more than ten Bragg peaks were marked as crystal hits. The fraction of diffraction images with crystal hits was reported during data collection on a real-time ‘hit rate’ plot. Diffraction patterns with marked Bragg spots and resolution rings were displayed at a refresh rate of 1 Hz for visual inspection of diffraction quality. After completion of each run, the pipeline applied corrections (pedestal subtraction, gain correction and bad pixel masking) to each raw diffraction image identified as crystal hit. Corrected diffraction images with pixel values in analog-to-digital units (ADU) (keV) were saved as separate HDF5 files for each run. The pipeline automatically invoked the *indexamajig* program from *CrystFEL* version 0.8.0 (White *et al.*, 2012 ▸) to index corrected diffraction patterns with crystal hits identified in the previous step. Indexing was performed with known unit-cell parameters using *MOSFLM* (Battye *et al.*, 2011 ▸) with peak-finding parameters, beam-center position and sample-to-detector distance optimized at the beginning of the experiment. The number of indexed patterns and the average resolution of found peaks for each run were automatically written to a shared spreadsheet. The Monte Carlo merging using *process_hkl* program from *CrystFEL* was initiated each time when an interval of 5000 indexed patterns was reached. Finally, merged Bragg spot intensities were converted to an MTZ file using *create-mtz* script from *CrystFEL*. Results from the well configured real-time SFX data-processing pipeline at SwissFEL were sufficient to judge the overall progress of the experiment. However, to obtain the best results, the data sets were re-processed after the experiment with carefully optimized parameters.

### 2.4. Off-line data processing, phasing and refinement   

The *CrystFEL* software-suite version 0.9.1 (White *et al.*, 2012 ▸) was used to analyze corrected diffraction images identified as crystal hits by the real-time SFX data-processing pipeline. The *indexamajig* program performed peak finding using the *peakfinder8* algorithm with threshold 70 for the nominal SASE and 170 ADU for the large-BW data sets with the minimal signal-to-noise ratio (SNR) = 5. Other peak-finding parameters were --min-pix-count = 2 and --local-bg-radius = 4. The geometry of the multi-segment JUNGFRAU 16M detector was refined using *geoptimizer* from *CrystFEL* (Yefanov *et al.*, 2015 ▸). The *detector-shift* script from *CrystFEL* was used to optimize the beam-center position. The sample-to-detector distance was optimized by finding the distance for which the standard deviation of the distribution of unit-cell parameters was the smallest, as described by Nass, Cheng *et al.* (2020 ▸) and Nass *et al.* (2016 ▸).

Off-line indexing of the nominal SASE and large-BW data sets was performed with the *XGANDALF* algorithm (Gevorkov *et al.*, 2019 ▸) with known unit-cell parameters. This indexing algorithm does not consider BW. The large-BW data set was also indexed using the *pinkIndexer* algorithm recently developed for processing pink-beam X-ray and electron-diffraction snapshots (Gevorkov *et al.*, 2020 ▸). A modified *CrystFEL* 0.9.1 version was used to process the large-BW data set with *pinkIndexer* (removed lines 865–876 from *integration.c* source-code file) to enable correct handling of an increased number of predicted reflections in *CrystFEL* arising from enlarged spectral width of the X-ray pulses. In addition, *CrystFEL* considers BW during spot prediction but not during prediction refinement, therefore it is disabled by *pinkIndexer* which uses its own prediction-refinement protocol that considers BW. The option --no-check-peaks, which disables *CrystFEL*’s check if indexing is successful, is automatically added when *pinkIndexer* is used. *PinkIndexer* performs this check internally considering BW. The *indexamajig* parameters used with this algorithm were --fix-profile-radius = 0.001e8, --pinkIndexer-tolerance = 0.04, --pinkIndexer-refinement-type = 4, --pinkIndexer-angle-resolution = 2, --pinkIndexer-considered-peaks-count = 4 and photon_energy_bandwidth = 0.015 defined in the geometry file.

Bragg peak intensity integration in *indexamajig* was performed using the *rings-grad* method for all data sets with the parameter --int-radius = 4, 5, 7. To avoid possible bias in the comparison of data-quality indicators between nominal SASE and large-BW data sets due to overlapping reflections from multiple crystals, we used *indexamajig* parameters --no-multi, --no-retry and --check-peaks for all data sets. Integrated reflection intensities were merged using *partialator* from the *CrystFEL* software-suite version 0.8.0 with partiality corrections and post-refinement (White *et al.*, 2016 ▸). Partiality corrections and post-refinement protocols currently implemented in *partialator* do not consider BW. The following parameters were used for merging in *partialator*: --model = xsphere, --iterations = 1, --no-deltacchalf and --push-res = 0.5. In addition, *partialator* from *CrystFEL* 0.9.1 performed worse than from *CrystFEL* 0.8.0 (Fig. S7 of the supporting information). The resulting set of merged intensities was converted to the XDS_ASCII format, passed through *XSCALE* for outlier rejection and converted to structure-factor amplitudes using the *XDSCONV* software package (Kabsch, 2010 ▸). The anomalous correlation coefficient (CC_ano_), SNR, *R*_split_ and the redundancy of individual *hkl* intensity measurements were calculated by *compare_hkl* and *check_hkl* programs from *CrystFEL*. The correlation coefficient (CC_anoref_) between the observed (|Δ*F*_obs_|) and expected (|Δ*F*_calc_|) anomalous difference structure-factor amplitudes was calculated using a custom script that is available upon request. The peak-finding threshold and SNR parameters in *indexamajig* were optimized to yield the highest indexing rates. In addition, the intensity-integration ring sizes were adjusted and various refinement types in *pinkIndexer* were tested to yield the highest overall CC_ano_ values. For *partialator*, the number of iterations (1, 2 or 3) and push-res values [infinity (the default) or 0.5] with and without --no-deltacchalf were optimized to yield the best *de novo* structure solution with the minimal number of indexed patterns for our sample and experimental conditions.

Structure determination by the native-SAD phasing method and automatic model building was performed using the *autoSHARP* pipeline version 2.8.12 (Vonrhein *et al.*, 2007 ▸). *AutoSHARP* searched for initial sulfur sites using *SHELXD* (Schneider & Sheldrick, 2002 ▸), which were then refined with *SHARP*. After phasing and solvent flattening with automated optimization of the solvent content, *autoSHARP* performed initial model building with *Buccaneer* (Cowtan, 2006 ▸). Automatic completion of the initial models was performed using *ARP/wARP* version 8 (Langer *et al.*, 2008 ▸) or the *Autobuild* module from *Phenix* (Adams *et al.*, 2010 ▸). Additionally, iterative cycles of manual model rebuilding in *Coot* (Emsley & Cowtan, 2004 ▸) and refinement using *Phenix* were performed to obtain final models. The model quality was assessed using *MolProbity* (Chen *et al.*, 2010 ▸).

### 2.5. X-ray pulse parameters at SwissFEL   

The X-ray pulses at SwissFEL (Prat, Abela *et al.*, 2020 ▸) were generated at a repetition rate of 25 Hz with an upper limit for the pulse duration of 30 fs (FWHM) (estimated from a measured electron-bunch length of 20 fs RMS). The pulses were focused by a pair of Kirkpatrick–Baez mirrors to ∼5 × 7 µm, measured by knife-edge scans at the sample position.

In the nominal SASE mode, the X-ray pulse parameters were as follows: a photon energy of 5.99 keV (2.069 Å) with an energy BW (Δ*E*/*E*) of 0.17% (Fig. S1) as measured by the photon single-shot spectrometer (PSSS) (Juranić *et al.*, 2018 ▸). The average pulse energy was 170 µJ as measured by the SwissFEL pulse intensity monitor (Juranić *et al.*, 2018 ▸). In the large-BW mode, the X-ray pulse parameters were as follows: a photon energy of 6.02 keV (2.059 Å) with an energy BW (Δ*E*/*E*) of 2.2% (Fig. S2) as measured by monochromator scans. The average pulse energy was 380 µJ. Using an estimated beamline transmission of ∼50%, and attenuation factors of 50% for the nominal SASE and 85% for the large-BW X-ray pulses, the pulse energy delivered to the sample was ∼35 to 40 µJ.

The large-BW X-ray pulses were generated at SwissFEL by maximizing the energy chirp and optimizing the compression scheme of the electron beam. By using this method, the X-ray BW can be continuously tuned up to ∼4% in the whole range of X-ray photon energies between 2 and 12.4 keV accessible at SwissFEL (Prat, Dijkstal *et al.*, 2020 ▸; Saa Hernandez *et al.*, 2016 ▸).

### 2.6. Simulations of diffraction patterns   

To simulate X-ray diffraction patterns, we used the *nanoBragg* program (https://bl831.als.lbl.gov/~jamesh/nanoBragg/). We used the geometry of the Alvra end station, specifically the dimensions of the JUNGFRAU 16M detector of ∼373.65 × 373.65 mm with square pixel size of 75 µm^2^, sample-to-detector distance of 95 mm, and X-ray photon energy of 4.5 keV with 3% and 3.5% that are within the capabilities of SwissFEL. With this setup, the maximal resolution at the edge of the diffraction patterns was ∼2.6 Å. We simulated diffraction patterns of the 5-HT_2B_ receptor crystal structure (Protein Data Bank; PDB entry 4nc3; Liu *et al.*, 2013 ▸). Parameters used for the simulations of the whole detector were -lambda 2.755, -dispersion 3.0 or 3.5, -detsize 373.65, -dispsteps 50, -pixel 0.075, -mosaic 0, 0.1, 0.2 or 0.3, -N 10, -water 0, -osc 0, -fluence 1.4e24, -tophat_spots and -nonoize. For simulations of the bottom-right quarter of the JUNGFRAU 16M detector we also used -detsize 150, -Xbeam 0 and -Ybeam 0. For simulations of the 1/16th of the detector in the bottom-right corner, we used -detsize 75, -Xbeam −75, -Ybeam −75, -fluence 1.4e34 and -mosaic_domains 50.

### 2.7. Data availability   

All data are available in the manuscript, the supporting information or at publicly accessible repositories. We deposited all diffraction images containing crystal hits used in this study, final *CrystFEL* stream and geometry files to the Coherent X-ray Imaging Data Bank (Maia, 2012 ▸) with the accession code ID 180 (https://cxidb.org/id-180.html). The atomic coordinates and structure-factor amplitudes were deposited in the PDB under the accession codes 7o44, 7o51, 7o53, 7o5J and 7o5K. The intermediate structures, log files and scripts are available from the corresponding authors upon request.

## 3. Results   

### 3.1. Experimental setup, X-ray beam and data-collection parameters   

To test whether large-BW XFEL pulses offer advantages for SFX when compared with pulses with smaller typical SASE XFEL BW postulated earlier (Dejoie *et al.*, 2013 ▸; White *et al.*, 2013 ▸), we used well diffracting thaumatin microcrystals. Here, we used the weak anomalous signal from endogenous sulfur atoms in thaumatin molecules as a sensitive data-quality indicator. We evaluated the data quality while systematically decreasing the number of indexed patterns, and determined the minimum for successful native-SAD phasing and automatic model building for each data set and indexing method (see *Methods* [Sec sec2]).

The SFX data sets from thaumatin microcrystals were measured at SwissFEL (Prat, Abela *et al.*, 2020 ▸) at the Alvra experimental station, see Section 4.1 of Milne *et al.* (2017 ▸). X-ray pulses with photon energy of 5.99 keV (2.069 Å) and nominal SASE energy BW (Δ*E*/*E*) of 0.17%, and with photon energy of 6.02 keV (2.059 Å) and large energy BW (Δ*E*/*E*) of 2.2%, were used to obtain two high-resolution thaumatin SFX data sets (SASE and large-BW data sets, respectively). Diffraction images were recorded at a repetition rate of 25 Hz with the low-noise automatic gain switching JUNGFRAU 16M detector (Mozzanica *et al.*, 2016 ▸) installed at the back of the Alvra Prime experimental chamber. Thaumatin microcrystals embedded in LCP were delivered to the focused X-ray pulses using a high-viscosity LCP injector (see *Methods* [Sec sec2] for more details).

In total, we recorded 350 000 images for the SASE data set, out of which 211 783 contained crystal hits identified by the real-time SFX data-processing pipeline (see *Methods* [Sec sec2]). After increasing the photon-energy BW, we recorded 710 000 images for the large-BW data set, out of which 225 346 contained crystal hits identified by the pipeline. The lower hit rate of the large-BW data set is due to lower concentration of the microcrystals embedded in LCP that was adjusted to reduce the probability of measuring multiple hits. The average per-shot photon energy of the SASE data set was 5992 eV with a standard deviation of 4 eV (Fig. S1). This small variation of the per-shot photon energy had negligible effect on the data-quality indicators of the SASE data set, therefore we used fixed photon energy to index all diffraction patterns from the SASE data set.

### 3.2. *De novo* structure determination of the SASE data set   

To perform an unbiased comparison excluding differences in experimental setups, sample characteristics and environmental conditions, we measured the SASE and large-BW data sets at the same XFEL facility during a single beam time while performing the best effort to maintain constant experimental parameters. Additionally, to avoid crystallographer’s bias, we used a black-box native-SAD phasing and model-building pipeline implemented in *autoSHARP* to determine the minimum number of images needed for phasing. The criterion for successful automatic *de novo* structure determination was more than 80% of built residues correctly placed in the amino acid sequence.

First, to establish the baseline for the evaluation of potential benefits of using large-BW X-ray pulses for SFX, we analyzed the thaumatin data set measured with 5.99 keV X-ray pulses with a nominal SASE BW of 0.17%. From this data set containing 211 783 hits, *CrystFEL* indexed 102 277 patterns (indexing rate of 48.3%) using the *XGANDALF* algorithm developed for indexing diffraction patterns obtained with small-BW radiation (Δλ/λ ≤ 5 × 10^−3^). The per-injector optimized detector distances were 95.50 and 96.36 mm. The maximal resolution of this data set was limited by the photon wavelength and geometrical setup to 2.0 Å.

For the whole SASE thaumatin data set with 102 277 indexed images and overall CC_ano_ of 0.153 (Table 1 ▸), *autoSHARP* determined the heavy-atom substructure and after phasing, density modification and model building, the final model consisted of 204 residues correctly placed in the protein sequence. This model was manually adjusted in *Coot* and refined in *Phenix*. Phasing details and data-quality indicators from *CrystFEL* are given in Tables S1 and S2 of the supporting information. The structure determination in *autoSHARP* with 90 000 indexed images did not result in an interpretable electron-density map. Therefore, we established that the baseline number of indexed images used for comparisons of data-quality indicators between the SASE and large-BW data sets is ∼102 000.

### 3.3. *De novo* structure determination of the large-BW data set indexed with *XGANDALF*   

Although the *XGANDALF* indexing algorithm was developed for indexing diffraction patterns obtained with small-BW radiation (Δ*E*/*E* ≤ 5 × 10^−3^), we tested its performance on the large-BW thaumatin data set measured with photon energy of 6.02 keV and 2.2% BW.

From 225 346 crystal hits identified by the real-time SFX data-processing pipeline, the *XGANDALF* algorithm indexed 130 621 patterns (indexing rate of 57.9%). The optimized detector distance for this data set was 95.0 mm. The maximal resolution of this data set was limited to 2.2 Å as indicated by the SNR of 1.38 and CC_1/2_ of 0.594 in the highest resolution shell between 2.20 and 2.25 Å (Table S3). Although we used thaumatin crystals from the same sample-preparation batch, we observed a lower resolution limit for this data set compared with the SASE data set. In particular, the SNR in the SASE data set in the 2.00–2.05 Å resolution shell is 4.42, almost three times higher than in this data set that has almost 28 000 more indexed images. We attribute this observation to the reduced SNR at high scattering angles due to the Bragg spot intensity dilution effect arising from thicker Ewald sphere when using large-BW X-rays for crystallography and to incorrect spot predictions due to uncertainty of the incident wavelength that gave rise to a given Bragg spot.

The heavy-atom substructure determination, phasing and automatic model building in *autoSHARP* as the structure determination with all indexed images was straightforward, owing to accurate measurement of the anomalous signal as indicated by the high overall CC_ano_ of 0.476 (Table S4). After phasing, density modification and automatic model building in *autoSHARP*, the final model consisted of 200 residues correctly placed in the protein sequence (Table S1).

For direct comparison of data-quality indicators and native-SAD phasing results of the large-BW data set indexed using *XGANDALF* with the SASE data set, we prepared the baseline large-BW data set with 102 000 randomly selected indexed patterns from the whole data set. Overall and split-into-resolution-shells statistics for this baseline large-BW data set are shown in Tables 1 ▸ and S5. Also for this data set, phasing and automatic model building in *autoSHARP* was undemanding. The final model consisted of 199 residues correctly placed in the protein sequence.

We systematically decreased the number of indexed patterns and determined that 50 000 indexed images from the large-BW thaumatin data set indexed with *XGANDALF* was the minimum for successful automatic native-SAD phasing and model building in *autoSHARP*. After phasing, density modification and automatic model building, the final model consisted of 204 residues correctly placed in the protein sequence. The overall and split-into-resolution-shells statistics for this data set are shown in Tables 1 ▸ and S6

This result shows a twofold decrease in the number of indexed images needed for automatic *de novo* structure determination of thaumatin when compared with the data set measured with a nominal SASE BW of 0.17% and indexed with *XGANDALF*. Consequently, we determined that similar precision of structure-factor amplitudes can be obtained in SFX with at least twofold reduction in sample and beam-time consumption using X-ray pulses with 2.2% BW when compared with the nominal SASE BW of 0.17%.

We performed ten trials in *autoSHARP* with resolution cut-offs between 2.2 and 4.5 Å using a data set with 40 000 indexed images selected randomly from the large-BW data set indexed by *XGANDALF* but the automatically built model did not contain enough residues placed in the amino acid sequence to satisfy our criteria for successful automatic structure determination in *autoSHARP*. After visual inspection of the models obtained from *autoSHARP* with 40 000 images, none of them resembled the shape of a globular molecule, hence it was unlikely that manual model re-building in *Coot* could yield the correct thaumatin structure. Therefore, we concluded that at least 50 000 indexed images from the large-BW thaumatin data set indexed with *XGANDALF* were needed for automatic structure determination in *autoSHARP*.

### 3.4. *De novo* structure determination of the large-BW data set indexed with *pinkIndexer*   

The main bottleneck in indexing pink-beam diffraction patterns by automatic indexing algorithms suited for monochromatic crystallography experiments (*e.g. XGANDALF*) is the uncertainty of the particular incident wavelength that gave rise to a given Bragg spot. This is particularly pronounced at high scattering angles where the diffraction conditions of many Bragg spots are far from the central wavelength and can contribute to the deterioration of the data quality at high resolution observed in the previous section. To overcome this problem, the *pinkIndexer* algorithm (Gevorkov *et al.*, 2020 ▸) was developed for automatic indexing of snapshot diffraction patterns obtained from serial pink-beam X-ray crystallography experiments. It was successfully used in Gevorkov *et al.* (2020 ▸) for processing polychromatic diffraction patterns acquired during serial crystallography experiments at synchrotrons (Meents *et al.*, 2017 ▸; Tolstikova *et al.*, 2019 ▸).

Here, we explored whether the *pinkIndexer* algorithm integrated into the *CrystFEL* data-processing suite allows obtaining accurate measurements of structure-factor amplitudes needed for *de novo* phasing with fewer diffraction images from the large-BW data set than *XGANDALF*. As with *XGANDALF*, we carefully optimized the *pinkIndexer* parameters to yield the best *de novo* structure-determination results and data-quality indicators with the least number of indexed images from the large-BW data set (see *Methods* [Sec sec2]). From 225 346 crystal hits, the *pinkIndexer* algorithm indexed 135 482 patterns (indexing rate of 60.1%). The maximal resolution of this data set was limited to 2.05 Å as indicated by the SNR of 1.16 and CC_1/2_ of 0.597 in the highest resolution shell between 2.05 and 2.10 Å (Table S7). The overall CC_ano_ of this data set indexed by *pinkIndexer* (CC_ano_ of 0.746) is higher than that of the same data set indexed by *XGANDALF* (CC_ano_ of 0.476). Additionally, the high-resolution limit of the large-BW data set processed with *pinkIndexer* (2.05 Å) is higher than that of the same data set processed with *XGANDALF* (2.2 Å) but not as high as for the SASE data set (2.0 Å). We attribute these improvements to *pinkIndexer*’s ability to utilize BW during indexing and prediction refinement, which *XGANDALF* lacks.

The heavy-atom substructure determination, phasing and automatic model building in *autoSHARP* of the large-BW thaumatin data set with all indexed images processed with *pinkIndexer* was straightforward, owing to very accurate measurement of the anomalous signal as indicated by the high overall CC_ano_ of 0.746 (Table S8). After phasing, density modification and automatic model building in *autoSHARP*, the final model consisted of 199 residues correctly placed in the protein sequence (Table S1).

To assess if the large-BW data set indexed with *pinkIndexer* is of better quality than the same data set indexed by *XGANDALF*, we compared data-quality indicators and native-SAD phasing results using data sets with equal numbers of indexed images. For this comparison, we prepared the baseline large-BW data set with 102 000 indexed patterns randomly selected from the whole data set. Overall and split-into-resolution-shells statistics for this data set calculated up to a resolution of 2.05 Å are shown in Tables 1 ▸ and S9 and up to a resolution of 2.2 Å in Tables S10 and S11. For the data set with 102 000 indexed images, phasing and automatic model building in *autoSHARP* was successful. After phasing, density modification and automatic model building, the final model consisted of 201 residues correctly placed in the protein sequence (Table S1).

Next, we systematically decreased the number of indexed patterns and determined the minimum for which automatic phasing and model building in *autoSHARP* was still successful. At least 30 000 indexed images from the large-BW data set indexed with *pinkIndexer* were needed for automatic structure determination of thaumatin in *autoSHARP*. The overall and split-into-resolution-shells statistics for this data set are shown in Tables 1 ▸ and S12, and up to a resolution of 2.2 Å in Tables S13 and S14. For the large-BW data set with 30 000 indexed images, the final model consisted of 200 residues with 167 of them correctly placed in the protein sequence (Table S1). With 25 000 indexed images selected randomly from this data set, automatic structure determination in *autoSHARP* failed in all trials. With only 30 000 indexed images needed for *de novo* structure determination and automatic model building, this result demonstrates ∼3.5-fold improvement when compared with the number of images from the SASE thaumatin data (∼102 000) and almost twofold improvement when compared with the number of images from the large-BW data set indexed with *XGANDALF* (50 000).

## 4. Discussion   

Large-BW XFEL pulses would in principle be beneficial for SFX by improving the partiality of individual reflections and increasing the number of Bragg spots per image (Dejoie *et al.*, 2013 ▸; White *et al.*, 2013 ▸). As demonstrated theoretically, these improvements lead to a faster convergence rate of SFX data sets in Monte Carlo integration. Therefore, pink-beam or large-BW SFX offers advantages not only for *de novo* structure determination from small and weakly diffracting crystals but also for other types of SFX experiments, *e.g.* time-resolved studies, by enabling accurate measurements of structure-factor amplitudes with reduced sample and beam-time consumption. Consequently, this method of data collection at XFELs might facilitate SFX to a larger community of researchers. In particular, structure determination of difficult to crystallize systems, *e.g.* GPCRs, is currently hindered by the need to grow large and well diffracting crystals that can withstand radiation damage at synchrotron sources. However, SFX allows essentially radiation-damage-free data collection from tiny crystals that tend to be better ordered and, in the case of GPCRs, typically diffract to higher resolution at XFEL sources than at synchrotrons (Stauch & Cherezov, 2018 ▸).

To provide an unbiased assessment, we performed a detailed comparison of the quality of SFX data sets obtained using nominal SASE and large-BW X-ray pulses at SwissFEL. We based the comparison on a very sensitive data-quality indicator available in our data sets: the weak anomalous signal from sulfur atoms (estimated Bijvoet ratio 〈|Δ*F*|〉/〈*F*〉 of ∼2% for thaumatin at 6 keV). We compared the anomalous correlation coefficient (CC_ano_), SNR, *R*_split_ and redundancy of individual *hkl* measurements as a function of resolution from data sets with the same (102 000) and minimal number of indexed images needed for automatic *de novo* structure determination.

### 4.1. Comparison of data-quality indicators between SASE and large-BW data sets with 102 000 images indexed using *XGANDALF* and *pinkIndexer*   

**Figure 1:**
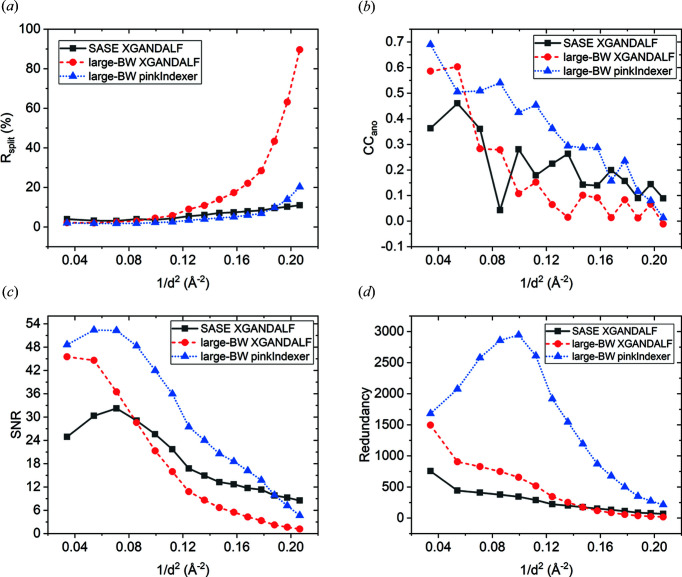
Comparison of data-quality indicators between the SASE and large-BW thaumatin data sets indexed by *XGANDALF* and *pinkIndexer* calculated with the same number of indexed images (102 000) up to a resolution limit of 2.2 Å. (*a*) *R*_split_, (*b*) CC_ano_, (*c*) SNR and (*d*) redundancy of individual reflections as a function of resolution.

For the comparisons, we used the maximal resolution limit of 2.2 Å for all data sets. The SASE data set has higher SNR and lower *R*_split_ factor in the high resolution shells between 2.3 and 2.2 Å than the large-BW data set with the same number of patterns indexed using *XGANDALF* or *pinkIndexer* (Fig. 1 ▸, and Tables S5, S11 and S15). As one of the contributing factors, we can explain the observed reduction of SNR and increase of *R*_split_ in the large-BW data set at high resolution by a smaller number of photons in a diffraction condition than for the SASE data set because we used similar average pulse energy at the sample position (see *Methods* [Sec sec2]). Essentially, the distance between the limiting Ewald spheres for large-BW X-ray pulses at high resolution is larger than the spectral width of the Bragg peaks, therefore smaller numbers of photons contribute to Bragg peaks and at the same time larger numbers of photons contribute to the background scattering, which results in lower SNR and higher *R*_split_.

The dilution of Bragg spot intensity at high resolution that arises from the thick Ewald sphere is one of the limitations of using pink or large-BW X-ray radiation for crystallography experiments at synchrotrons. The SNR at high resolution in pink-beam serial synchrotron crystallography experiments can be improved by using setups optimized for reducing background scattering (Meents *et al.*, 2017 ▸) but, due to the general pink-beam dilution effect that reduces SNR at high resolution, more photons per exposure are required to obtain similar diffraction-signal levels as with monochromatic radiation, especially for weakly diffracting micro-crystals. However, for typical synchrotron data-collection time scales, the destructive effects of global radiation damage that occur above certain absorbed dose limits (Garman & Weik, 2017 ▸) hinder the achievable maximal resolution. One way to partially overcome this problem is to spread the absorbed dose over multiple crystals *e.g.* in a serial synchrotron crystallography experiment. Unfortunately, this approach has severe limitations for pink-beam synchrotron experiments when only small micro-crystals are available or needed [*e.g.* in time-resolved studies where the intensity of the pump laser traveling throughout the crystalline material decreases exponentially (Grünbein *et al.*, 2020 ▸)]. This is because negative effects of radiation damage increase with the number of photons per exposure while the SNR at high resolution decreases with larger X-ray BW. Fortunately, in SFX most of the radiation-damage processes that affect synchrotron data collection are mitigated by femtosecond X-ray pulses (Nass, 2019 ▸). This allows for increasing the intensity of large-BW X-ray pulses to obtain better SNR at high resolution from small micro-crystals than at synchrotrons without the negative effects of radiation damage.

At low resolution the SNR of the large-BW data set indexed by *XGANDALF* and *pinkIndexer* is higher than of the SASE data set (Fig. 1 ▸), which can also be explained by a larger spectral coverage of the strong low-resolution reflections and consequently higher precision of the measurements of structure-factor amplitudes at low resolution. The higher CC_ano_, CC_anoref_ and lower *R*_split_ values for the large-BW data set at low resolution (Figs. 1 ▸ and S3) also indicate this. As expected from theoretical considerations, the redundancy of individual measurements is the largest for the large-BW data set (Fig. 1 ▸). We also observe that for the large-BW data set processed with *pinkIndexer*, the maximal resolution limit, SNR, *R*_split_, CC_1/2_ and CC_ano_ are higher than when using *XGANDALF* (Fig. 1 ▸, and Tables 1 ▸, S5, S10 and S11). This validates that *pinkIndexer* is indeed better suited for indexing diffraction patterns obtained with large-BW X-ray radiation than *XGANDALF*.

### 4.2. Comparison of data-quality indicators between SASE and large-BW data sets with minimal number of indexed images needed for *de novo* structure determination   

**Figure 2:**
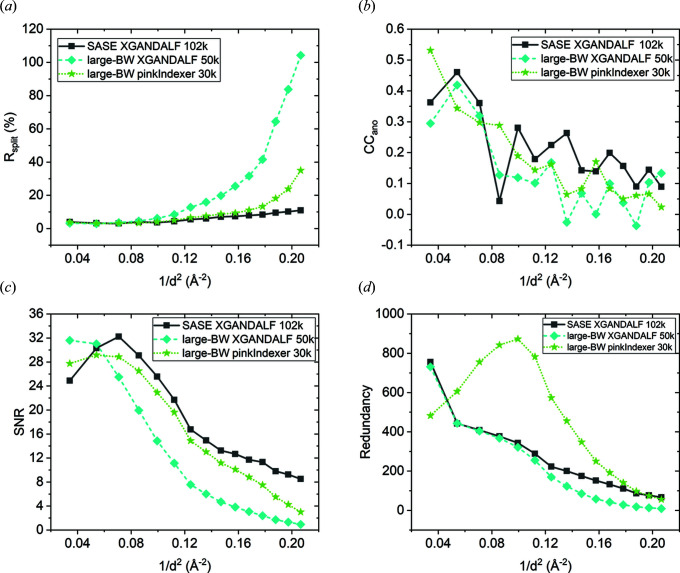
Comparison of data-quality indicators between the SASE and large-BW thaumatin data sets indexed by *XGANDALF* and *pinkIndexer* calculated with the minimal number of indexed images required for automatic *de novo* structure determination up to a resolution limit of 2.2 Å. (*a*) *R*_split_, (*b*) CC_ano_, (*c*) SNR and (*d*) redundancy of individual reflections as a function of resolution.

The minimal numbers of indexed images needed for automatic *de novo* structure determination from the SASE and large-BW thaumatin data sets indexed by *XGANDALF* and *pinkIndexer* were 102 000, 50 000 and 30 000, respectively. We calculated various data-quality indicators as functions of resolution for these data sets using the same high-resolution limit of 2.2 Å (Figs. 2 ▸ and S3, and Tables S6, S13, S14 and S15). Fig. 2 ▸ shows that *R*_split_ and SNR for the large-BW *XGANDALF* data set with 50 000 images are of lower quality than for the large-BW *pinkIndexer* data set with 30 000 images or for the SASE data set with 102 000 images. The CC_ano_ and CC_anoref_ are similar in the low-resolution range for all three data sets and decrease in higher resolution shells.

Interestingly, the *R*_split_, CC_anoref_ and SNR of the large-BW *pinkIndexer* data set with 30 000 images are similar to the SASE data set with 102 000 images and deteriorate only at high resolution. This observation might be attributed to the higher redundancy and improved partiality of individual reflections in the large-BW data set indexed by *pinkIndexer*, and the effects associated with the thicker Ewald sphere discussed above.

### 4.3. Comparison of the data-quality indicators for SASE and large-BW data sets with minimal number of indexed images needed for a complete set of accurate structure-factor amplitudes   

**Figure 3:**
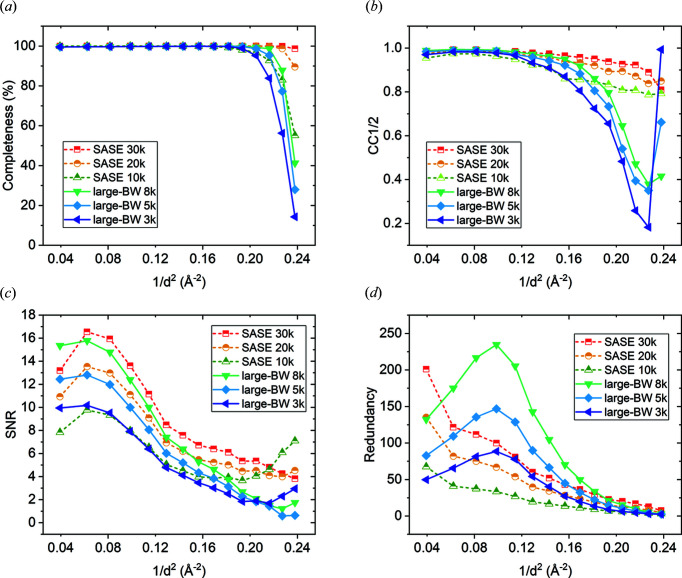
Comparison of data-quality indicators between the SASE and large-BW thaumatin data sets with various number of indexed images calculated up to a resolution limit of 2.05 Å. (*a*) Completeness, (*b*) CC_1/2_, (*c*) SNR and (*d*) redundancy of individual reflections as a function of resolution.

To investigate whether large-BW X-ray pulses can also be beneficial for other SFX applications, where molecular replacement is used for structure determination that does not require as high accuracy as native SAD, we calculated and compared data-quality indicators with smaller numbers of indexed images from the SASE and large-BW data sets (Fig. 3 ▸). To obtain high completeness (>80%), CC_1/2_ (>0.8) and SNR (>3) at high resolution from the SASE data set we needed at least 20 000 indexed images. In contrast, from the large-BW data set indexed using *pinkIndexer*, SNR values similar to the SASE data set with 20 000 images can be obtained with only 5000 indexed images, except for the high resolution shells. We attribute the deterioration of CC_1/2_, completeness and SNR at high resolution in the large-BW data set to the diffraction dilution effects discussed above and postulate that it can be overcome by increasing the intensity of large-BW XFEL pulses without negative global radiation-damage effects. The high-dynamic range of the JUNGFRAU detector used in this experiment would allow larger pulse intensity without observing overloads (Fig. S4). Therefore, if the large-BW X-ray pulses in our study had higher intensity we could achieve similar quality data also at high resolution with 5000 large-BW images as with 20 000 SASE images. Detailed comparison of *R*_split_ and CC_1/2_ as functions of the number of indexed diffraction patterns and resolution for various numbers of indexed patterns from the SASE and large-BW data sets is shown in Figs. S5 and S6.

### 4.4. Simulations of diffraction patterns from a typical GPCR crystal structure   

Radially streaked and overlapping Bragg spots in diffraction patterns measured from macromolecular crystals with high mosaicity or with very large unit cells are one of the limitations of polychromatic Laue X-ray crystallography at synchrotrons (Ren & Moffat, 1994 ▸; Shrive *et al.*, 1990 ▸). Although methods to extract individual Bragg spot intensities from overlapping reflections measured at synchrotrons exist (Shrive *et al.*, 1990 ▸), they are unpractical for serial crystallography where thousands of still images are measured from crystals of different sizes with no orientation relation between consecutive images. In this study, we used well ordered thaumatin crystals with very small mosaicity and relatively small unit-cell constants. Additionally, the large-area JUNGFRAU 16M detector and the low photon energy of 6 keV decreased the probability of observing overlaps. However, for SFX experiments with large-BW X-ray pulses, overlapping reflections from crystals with high mosaicity and large unit cells might be problematic.

**Figure 4:**
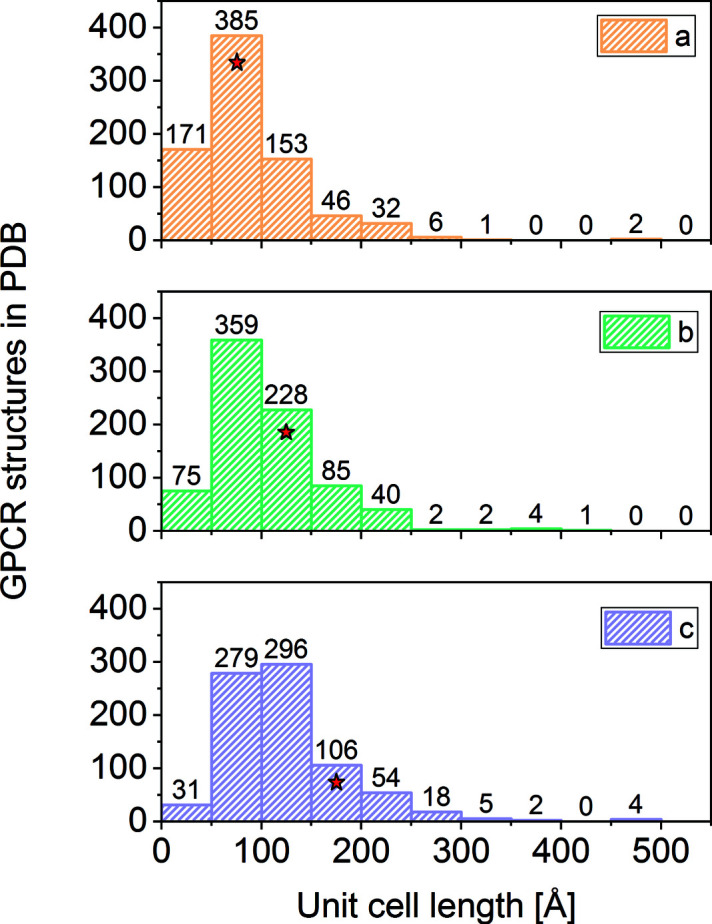
Histograms of the unit-cell lengths for all unique GPCR entries deposited in the PDB (Berman *et al.*, 2003 ▸). Numbers above the bars indicate the count in each bin. The unit-cell lengths of the 5-HT_2B_ receptor belong to one of the most frequently occurring GPCR unit-cell lengths in the PDB (bins marked with the star) and therefore serve as a representative candidate for the simulations.

To investigate the effects of realistic mosaicity in SFX with large-BW X-ray pulses, we simulated diffraction patterns using the *nanoBragg* program (see *Methods* [Sec sec2]) for various mosaicity and X-ray BW settings using experimental geometry similar to the Alvra end station at SwissFEL. To focus on GPCR structure determination, as one of the most promising applications of SFX, we used the crystal structure of the 5-HT_2B_ receptor determined via SFX to a resolution of 2.8 Å (PDB entry 4nc3) with unit-cell lengths of *a* = 61.5, *b* = 122.2 and *c* = 168.5 Å (Liu *et al.*, 2013 ▸). We selected this PDB entry as an representative example of published GPCR crystal structures based on the distribution of unit-cell lengths of unique GPCR entries deposited in the PDB (Berman *et al.*, 2003 ▸) (Fig. 4 ▸).

**Figure 5:**
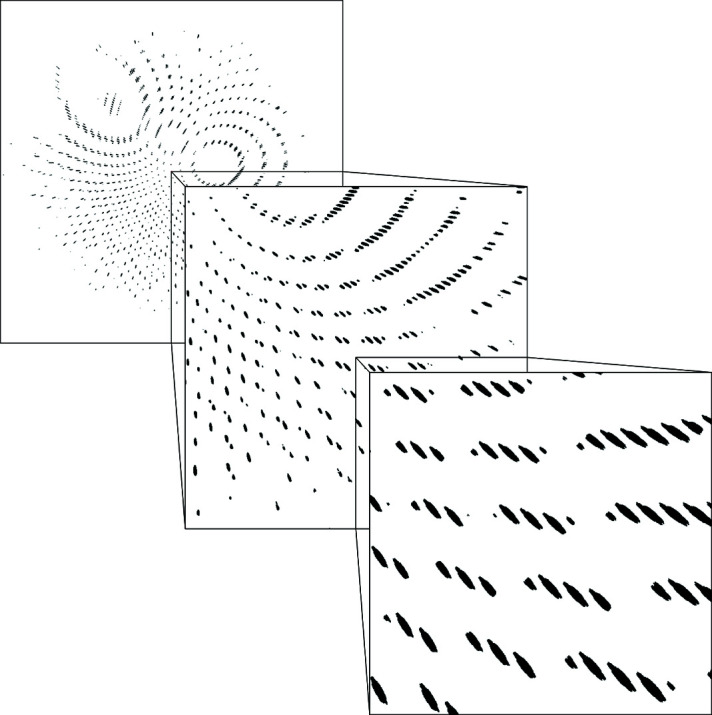
Simulated snapshot diffraction patterns from a randomly oriented crystal of the 5-HT_2B_ receptor (PDB entry 4nc3) with mosaicity of 0.2°. The simulations were performed with an X-ray BW of 3.5% (top-hat shape), photon energy of 4.5 keV and experimental geometry similar to the SFX setup at the Alvra end station at SwissFEL with the JUNGFRAU 16M detector. The resolution at the edge of the image is 2.6 Å.

**Figure 6:**
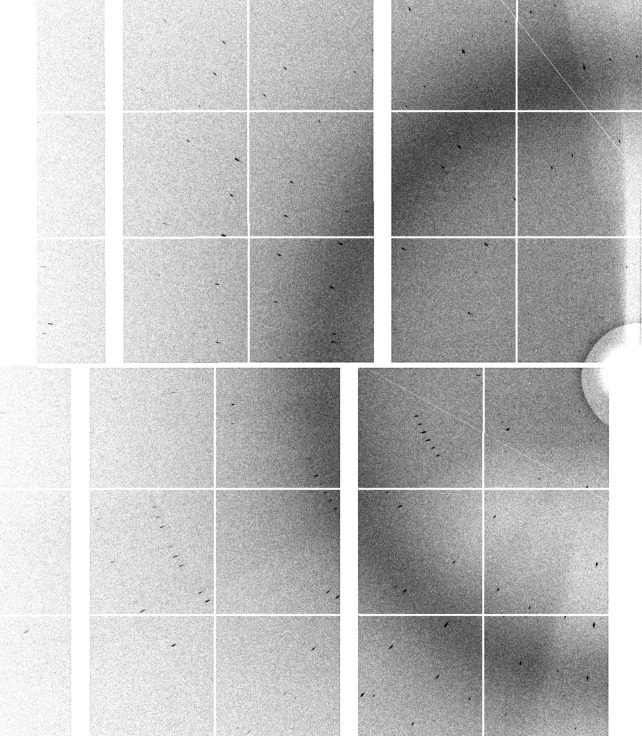
A fragment of a snapshot diffraction pattern from single thaumatin microcrystal recorded using the JUNGFRAU 16M detector and a large-BW X-ray pulse (Δλ/λ = 2.2% FWHM) from SwissFEL.

As an example, Fig. 5 ▸ shows simulated diffraction patterns from a randomly oriented crystal of the 5-HT_2B_ receptor with mosaicity of 0.2° calculated using 3.5% X-ray pulse BW and a photon energy of 4.5 keV. This simulated result demonstrates that sufficient separation between neighboring Bragg spots for intensity integration can be achieved in the whole achievable resolution range of a typical GPCR crystal with realistic mosaicity and average unit-cell lengths when the X-ray pulse parameters are tuned to specific values predicted by the simulations that are within the capabilities of SwissFEL. This opens up the opportunity for using more than one order of magnitude larger X-ray BW than the typical SASE BW for SFX with low photon energy and a large area detector with small pixel size. Simulated diffraction patterns with crystal mosaicity between 0 and 0.3° and X-ray BW of 3 and 3.5% can be found in Figs. S8–S15. Examples of thaumatin diffraction patterns measured with large-BW and SASE X-ray pulses at SwissFEL are shown in Fig. 6 ▸ and in the supporting information.

## 5. Summary and outlook   

By using large-BW X-ray pulses, we were able to reduce the number of images needed for obtaining an accurate set of complete structure-factor amplitudes in SFX experiments by more than a factor of three in comparison with using X-ray pulses with nominal SASE BW. We attribute this achievement to improved partiality of individual reflections and increased number of Bragg spots per image obtained with large-BW (pink) X-ray pulses used in combination with the new indexing algorithm developed for processing snapshot polychromatic diffraction patterns from crystals in random orientations. To obtain these results, we measured two large SFX thaumatin data sets using the same experimental conditions at SwissFEL with the only changes between measurements being the average spectral BW of the X-ray pulses (0.17 and 2.2%). Moreover, we performed detailed comparisons of data-quality indicators (*R*_split_, CC_ano_, CC_anoref_, CC_1/2_, SNR and completeness) calculated for data sets with the same number of indexed images and determined the minimum needed for automatic *de novo* structure determination via the native-SAD phasing method for each data set.

We deliberately used a model system and employed the weak anomalous signal from endogenous sulfur atoms to be able to provide unbiased assessment of the data-set accuracy between SASE and large-BW data sets. Additionally, we determined the minimal numbers of images from the two data sets needed for obtaining high-quality data sets for typical SFX applications that do not require as high accuracy as native SAD, such as time-resolved SFX and structure determination via molecular replacement. We found that the minimal number of images needed for a good quality data set could be reduced by a factor of four when using X-ray pulses with a large BW of 2.2% when compared with X-ray pulses with a nominal SASE BW of 0.17% and similar experimental conditions.

This makes the method of pink-beam SFX very well suited not only for cases where a limited amount of sample is available (*e.g.* GPCR structure determination) but also when in general it is difficult to obtain large high-quality crystals for structure determination at synchrotrons (*e.g.* membrane proteins) and only microcrystals are available. In addition, with the significant reduction in the number of indexed images needed for a high-quality data set and the development of automatic real-time data-processing pipelines, this method enables efficient usage of beam time and thus facilitates SFX to a larger community of researchers without extensive SFX experience.

X-ray pulses with increased spectral BW can be produced at other XFEL facilities and are not unique only to SwissFEL; however, because of the SwissFEL accelerator design with specific components that preserve the electron-beam quality after electron-bunch compression, SwissFEL can produce X-ray pulses with the largest energy spread. Moreover, the large energy spread is easier to achieve at low photon energies, which then requires SFX instrumentation optimized for data collection with long wavelengths such as He or vacuum environments and large-area X-ray detectors.

Upgraded third-generation synchrotron sources will provide ∼100 times larger average photon flux density in pink-beam modes. However, even with the shortest exposure times available at synchrotrons (∼100 ps), the radiation-damage effects caused by primary damage mechanisms cannot be outrun. The absorbed dose limits established for room-temperature serial synchrotron crystallography experiments (de la Mora *et al.*, 2020 ▸; Ebrahim *et al.*, 2019 ▸) can be easily exceeded at upgraded synchrotron sources during experiments with small, weakly diffracting and radiation-sensitive microcrystals. In contrast to room-temperature pink-beam serial crystallography at synchrotrons, obtaining high-quality data from small and weakly diffracting microcrystals with large-BW SFX at XFEL sources is not limited by radiation damage. Additionally, the optimal photon energy and BW of the XFEL pulses for a given crystal system can be easily estimated with simulations before the experiment and tuned at the beamline to achieve sufficient Bragg spot separation and resolution. This, in combination with the new pink-beam indexing software that is fully automatic and can deal with multiple crystals per pattern, suggests that pink-beam SFX might become the mainstream method for measuring accurate structure-factor amplitudes from small micro-crystals with reduced sample and beam-time consumption at XFEL facilities. In addition to indexing, other aspects of serial crystallography data-processing software such as partiality corrections and post-refinement protocols should be updated to include wider X-ray BW. Further improvements could be achieved by using better Bragg spot prediction and integration methods that are able to resolve spot overlaps and model Bragg spot shapes and the intensity of each pixel contributing to a Bragg spot by taking into account the crystal and X-ray pulse properties, such as per-shot X-ray spectra (Mendez *et al.*, 2020 ▸). Moreover, a promising method has been proposed for obtaining sub-femtosecond time resolution in pump–probe SFX experiments with polychromatic hard X-ray FEL pulses with an energy chirp available at SwissFEL (Fadini *et al.*, 2020 ▸).

In conclusion, we have demonstrated that XFEL-based pink-beam SFX is not only feasible but also advantageous. Using a well characterized model system and weak anomalous signal, we showed that significantly fewer indexed patterns are required for *de novo* phasing and for obtaining sufficiently accurate sets of complete structure factors for general SFX applications using large-BW X-ray pulses compared with nominal SASE BW. The reduction of the required amounts of sample and beam time offered by pink-beam SFX, as well as the automatic SFX data-processing pipeline developed at SwissFEL combined with experienced beamline staff, is expected to improve the accessibility of XFELs for unexperienced SFX users. We expect pink-beam SFX data collection to be particularly useful for SFX projects where it is difficult to obtain large amounts of sample typically required for SFX or where only tiny microcrystals are available that are too small for structural studies at synchrotron sources.

## Supplementary Material

Supporting information. DOI: 10.1107/S2052252521008046/zf5018sup1.pdf

PDB reference: Structure of thaumatin determined at SwissFEL using native SAD at 5.99 keV with photon-energy BW of 0.26%, 7o44

PDB reference: Structure of thaumatin determined at SwissFEL using native SAD at 6.02 keV with photon-energy BW of 2.15% and *pinkIndexer* with 30 000 indexed images, 7o5k

PDB reference: Structure of thaumatin determined at SwissFEL using native SAD at 6.02 keV with photon-energy BW of 2.15% and *XGANDALF*, 7o51

PDB reference: Structure of thaumatin determined at SwissFEL using native SAD at 6.02 keV with photon-energy BW of 2.15% and *pinkIndexer*, 7o5j

PDB reference: Structure of thaumatin determined at SwissFEL using native SAD at 6.02 keV with photon-energy BW of 2.15% and *XGANDALF* with 50 000 indexed images, 7o53

## Figures and Tables

**Table 1:** SFX data-collection and refinement statistics

Data set	SASE (*XGANDALF*)	Large BW (*XGANDALF*)		Large BW (*pinkIndexer*)	
	Baseline	Baseline	50 000	Baseline	30 000
Wavelength	2.069 Å/5.99 keV	2.059 Å/6.02 keV			
Bandwidth Δ*E*/*E* (%)	0.17	2.2			
Temperature (K)	297				
Space group	*P*4_1_2_1_2				
Cell dimensions *a*, *b*, *c* (Å)	58.52, 58.52, 151.30				
Number of collected patterns	350 000	710 000			
Number of hits (% of collected)	211 783 (60.5)	225 346 (31.7)			
Number of indexed patterns (% of hits)	102 277 (48.3)	102 000	50 000	102 000	30 000
Number of unique reflections	33 976	25 539	25 539	31 565	31 565
Resolution range (Å)	24.8–2.0	24.8–2.2	24.8–2.2	25.25–2.05	25.25–2.05
Completeness (%)	100 (99.8)	100 (99.9)	100 (96.5)	100 (99.8)	100 (92.0)
Average redundancy	200 (11)	418 (19)	205 (9)	1218 (30)	356 (8)
CC_1/2_	0.993 (0.869)	0.999 (0.501)	0.998 (0.424)	0.999 (0.426)	0.997 (0.218)
CC†	0.998 (0.964)	0.999 (0.817)	0.999 (0.772)	0.999 (0.773)	0.999 (0.600)
CC_ano_	0.153 (0.145)	0.492 (0.010)	0.284 (0.133)	0.670 (0.002)	0.509 (0.078)
Overall *I*/σ(*I*)	15.09 (4.42)	15.82 (1.18)	11.09 (0.94)	23.12 (1.01)	12.90 (1.02)
*R*_split_ (%)‡	5.10 (27.30)	3.80 (89.62)	5.49 (104.20)	2.43 (100.60)	4.58 (151.50)
Refinement					
Resolution (Å)	24.73–2.00	23.14–2.20	24.73–2.20	23.14–2.05	23.14–2.05
Number of reflections used in refinement	18 561	14 057	14 017	17 261	16 369
*R*_work_/*R*_free_	0.1430/0.1755	0.1528/0.1966	0.1564/0.1941	0.1571/0.1738	0.1610/0.1919
Number of atoms	1712	1614	1615	1632	
Protein	1558	1558	1558	1558	1558
Ligand	10	10	10	10	10
Water	144	46	47	64	78
Overall *B* factor (Å^2^)	28.4	49.0	49.4	48.0	45.4
R.m.s. deviations					
Bond lengths (Å)	0.011	0.010	0.009	0.008	0.009
Bond angles (°)	1.664	1.130	1.086	0.964	0.995
Ramachandran plot (%)					
Most favored	97.07	96.10	97.07	96.59	97.07
Additionally allowed	2.93	3.90	2.44	3.41	2.93
Disallowed	0	0	0.49	0	0

† Numbers in parentheses refer to the highest resolution shell.

‡ *R*_split_ = 2^−1/2^∑|*I*_even_ − *I*_odd_|/[(1/2)∑(*I*_even_ + *I*_odd_)].
