# Supplementary material for: Pink-beam serial femtosecond crystallography for accurate structure-factor determination at an X-ray free-electron laser
Source: IUCrJ. 2021 Sep 23;8(Pt 6):905–20. doi: 10.1107/S2052252521008046 (PMC8562661; doi:10.1107/S2052252521008046)

IUCrJ

Volume 8 (2021)

Supporting information for article:

Pink-beam serial femtosecond crystallography for accurate structure-factor determination at an X-ray free-electron laser

Karol Nass, Camila Bacellar, Claudio Cirelli, Florian Dworkowski, Yaroslav Gevorkov, Daniel James, Philip J. M. Johnson, Demet Kekilli, Gregor Knopp, Isabelle Martiel, Dmitry Ozerov, Alexandra Tolstikova, Laura Vera, Tobias Weinert, Oleksandr Yefanov, Jörg Standfuss, Sven Reiche and Christopher J. Milne

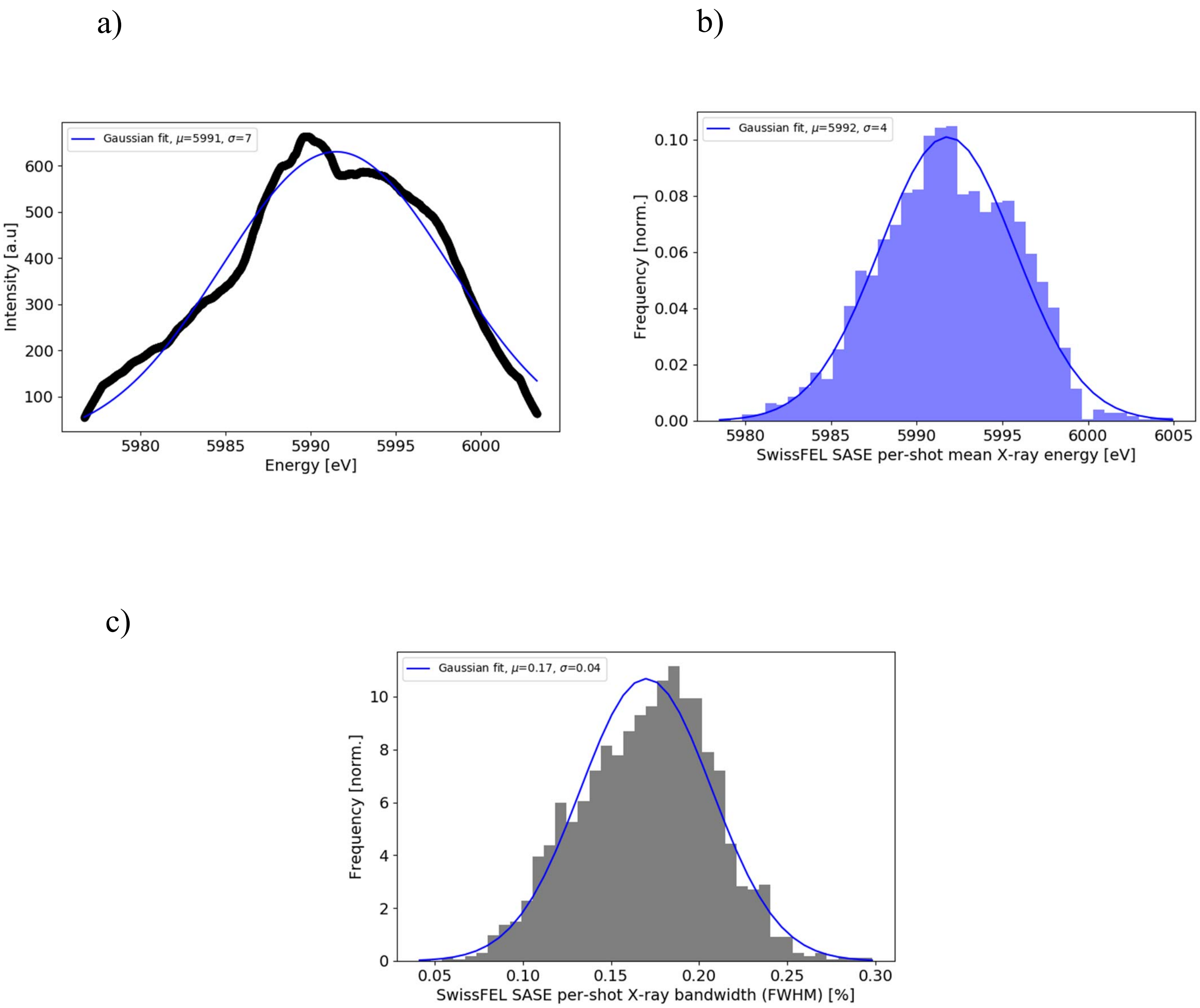

Figure S1 a) Average photon spectra of the nominal SASE X-ray pulses from 10,000 shots measured by the photon single-shot spectrometer (PSSS) at SwissFEL (black line). The mean photon energy estimated by the Gaussian fit (blue line) is 5991 ± 7 eV. b) Histogram of the mean photon energy estimated by fitting single-shot spectra from a). The mean photon energy estimated by the Gaussian fit (blue line) is 5992 ± 4 eV. c) Histogram of the SASE single-shot X-ray bandwidths (FWHM) estimated from fit parameters according to the formula $BW_i^{(FWHM)} = (2.355*\sigma_i)/\mu_i$ where i is the shot-number. The mean spectral bandwidth ($\Delta E/E_{SASE}$) of the SASE X-ray pulses estimated by the Gaussian fit (blue line) is 0.17 ± 0.04 %.

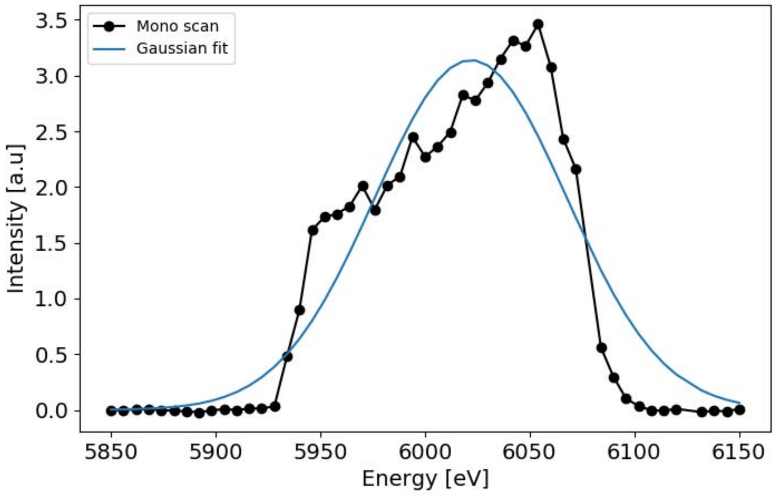

Figure S2 Photon spectra of the large-bandwidth X-ray pulses generated at SwissFEL obtained by scanning the monochromator and averaging the intensity of 500 pulses per step (black line and points). The parameters of the Gaussian fit (blue line) were as follows: mean = 6021.9 eV, standard deviation = 45.8 eV. The bandwidth estimated from the Gaussian fit was $\Delta E/E_{large\text{-}BW}$ ~1.8 %. However, because the Gaussian fit does not match the shape of the spectra (more top hat like), we estimated $\Delta E/E_{large\text{-}BW}$ ~ 2.2 % based on full width at half-maximum (FWHM) value read out from the plot.

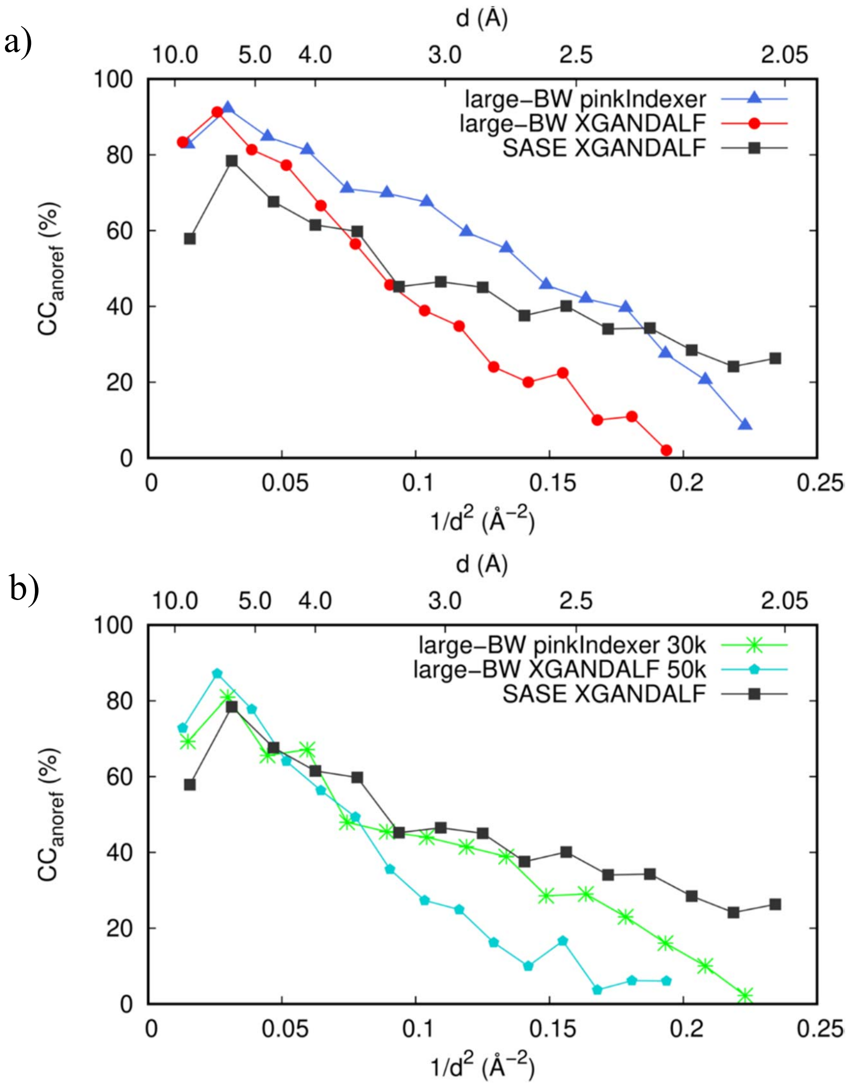

Figure S3 a) Correlation coefficient between the measured and calculated anomalous difference structure-factor amplitudes (CC_{anoref}) for the baseline thaumatin data sets with 102,000 indexed diffraction images (see Table 1 in the main text) and b) with the minimal amount of data necessary for successful structure solution using native-SAD. In the inset a), the large-BW data indexed with pinkIndexer has higher CC_{anoref} compared to the same large-BW data indexed with XGANDALF, which deteriorates quickly above resolution of ~4 Å despite having the same number of indexed images. In addition, the CC_{anoref} of the SASE data set indexed with XGANDALF is lower than for the large-BW data set indexed with pinkIndexer. In the inset b), the CC_{anoref} of the large-BW data set with 30k indexed images is comparable to CC_{anoref} of the SASE data set with ~102,000 indexed images. This indicates almost 3.5-fold improvement to achieve similar accuracy of structure factors as with SASE.

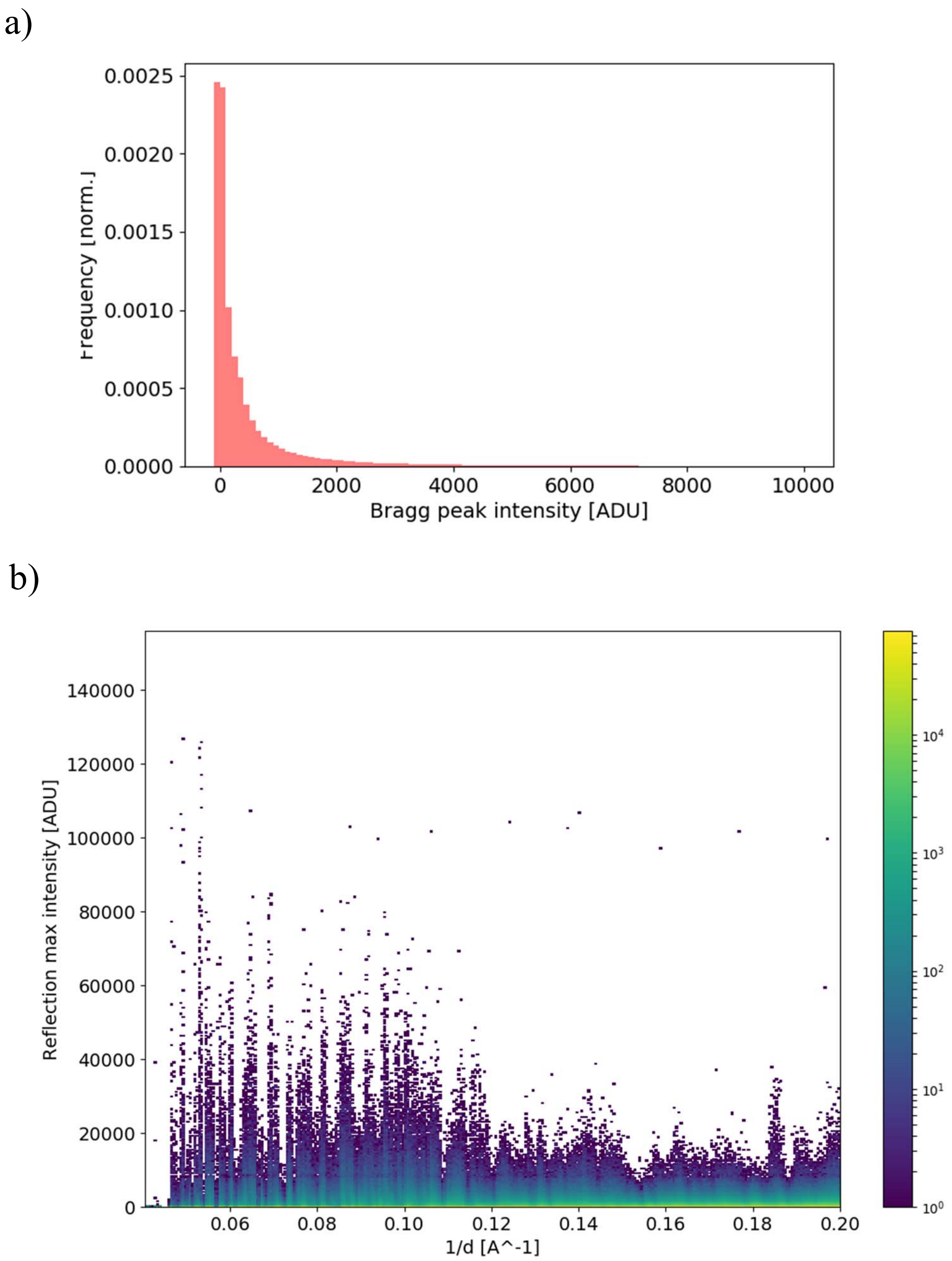

Figure S4 a) Histogram of the maximal pixel intensity of all found and predicted Bragg spots in the large-BW data set indexed by *pinkIndexer*. The mean count per peak is 649.32 ADU. The saturation limit for the JUNGFRAU detector is approximately 125,000 ADU. b) Histogram of the low-resolution reflections intensity created using the *peakogram-stream* script from *CrystFEL* (https://www.desy.de/~twhite/crystfel/tutorial.html#saturation). The majority of low-resolution reflections in the large-BW data set did not reach the detector saturation limit.

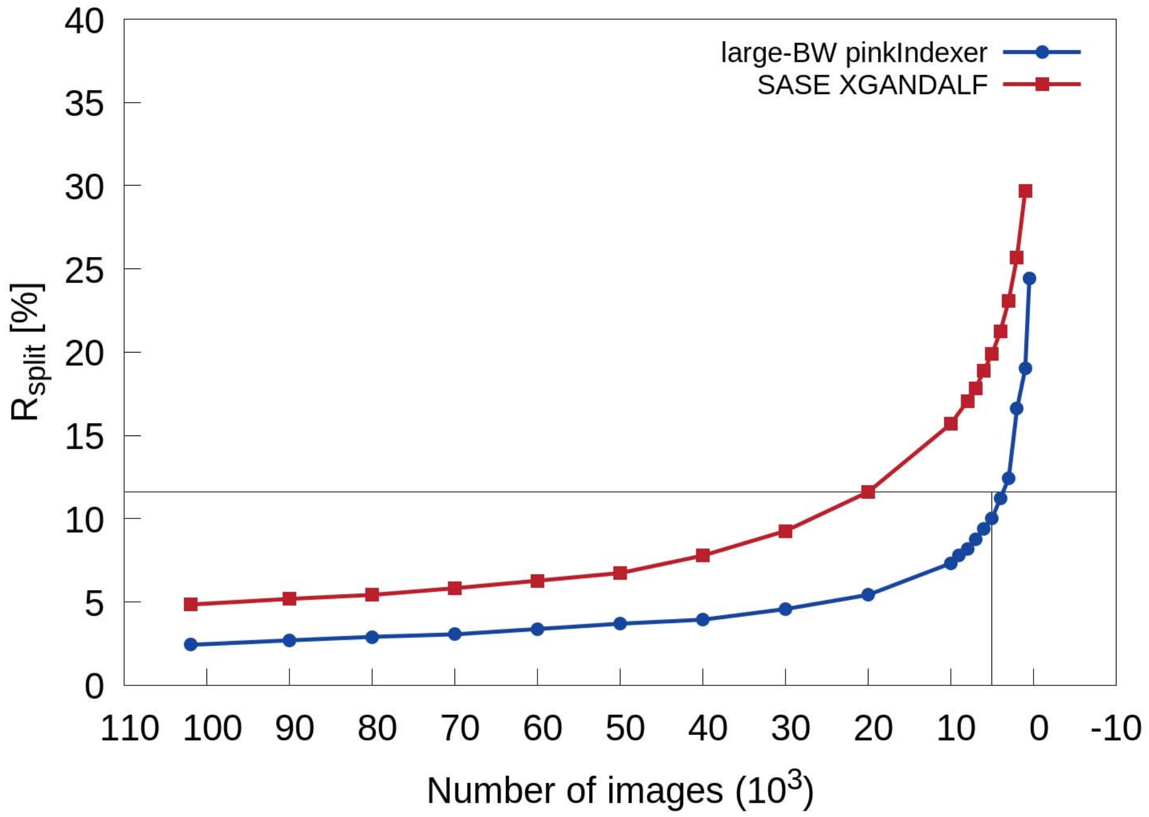

Figure S5 The overall R_{split} values calculated for the whole resolution range in function of the number of indexed diffraction patterns for the large-BW data set indexed with *pinkIndexer* (blue dots) and the SASE data set indexed with *XGANDLAF* (red squares). The horizontal line indicates overall R_{split} value of 11.60 % obtained with 20,000 SASE patterns and the vertical line indicates overall R_{split} value of 10.04 % obtained with 5,000 large-BW patterns. This suggests that ~4x fewer diffraction patterns are sufficient to obtain similar overall data quality with large-BW XFEL pulses when compared to SASE.

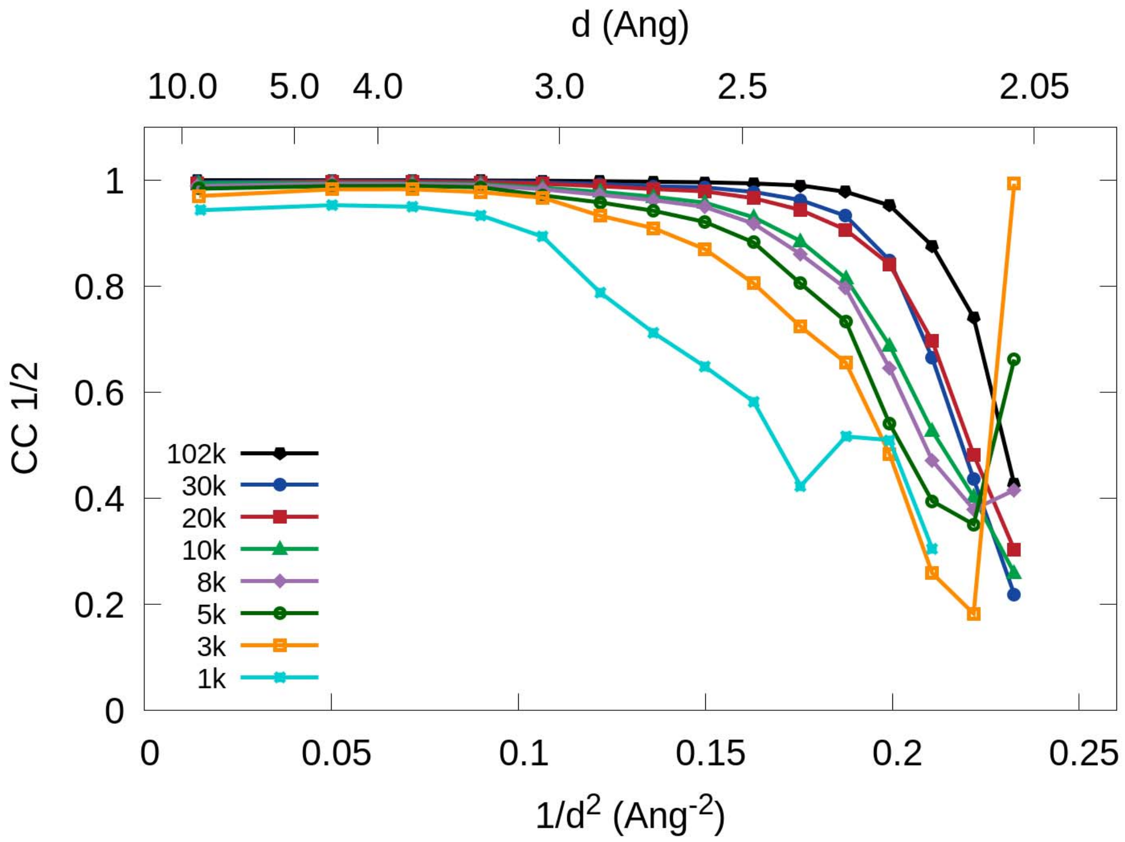

Figure S6 The CC 1/2 values in function of resolution for the large-BW data sets indexed with *pinkIndexer* and merged with various number of randomly selected diffraction images. The figure includes data sets that did not fit into Figure 3b in the main text due to readability.

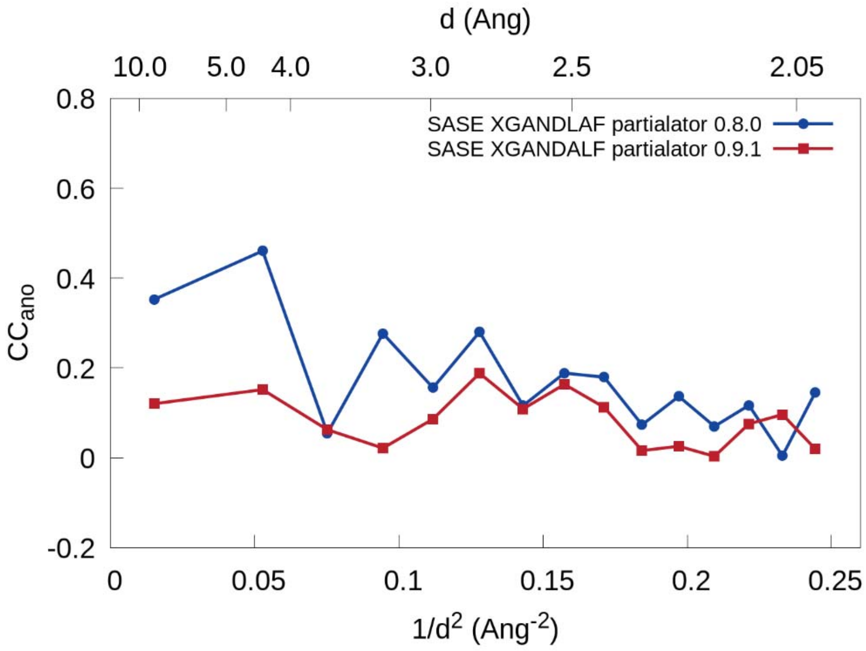

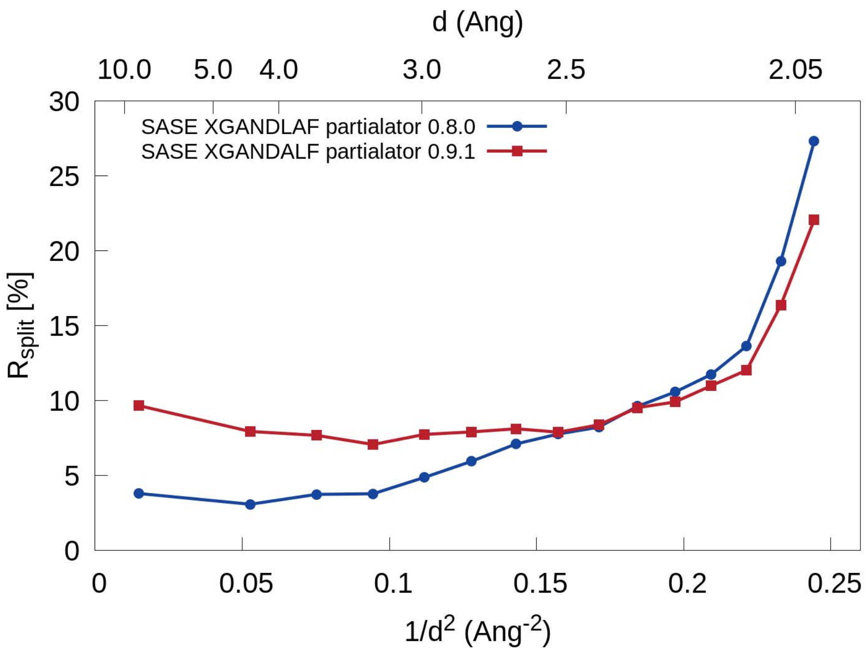

Figure S7 Comparison of CC_{ano} and R_{split} values in function of resolution for the thaumatin SASE data set indexed with *XGANDALF* and merged using *partialator* from CrystFEL 0.8.0 and 0.9.1 with partiality correction and post-refinement. It shows that *partialator* from CrystFEL 0.8.0 performed worse than 0.9.1 in the low and medium-high resolution.

Table S1 Phasing and automatic model building results from *autoSHARP* for the SASE and large-BW data sets.

	SHELXD					Initial model building with *BUCCANEER*			Final Model building with *ARP/Warp*			
Data set name	Resolution for HA substructure search (Å)	Number of initial HA sites	CFOM	CC	CC (weak)	Number of residues built	Number of chains	Number of residues placed in the sequence	Number of residues built	Number of chains	Number of residues placed in the sequence	R/R_{free} factors
SASE 102k XGANDALF	3.4	17	46.16	36.13	10.03	205	12	179	204	1	204	0.1704/ 0.2150
Large-BW 130k XGANDALF	2.2	17	72.04	52.30	19.73	185	12	164	200	3	200	0.1633/ 0.2170
Large-BW 102k XGANDALF	2.2	4	72.15	51.64	20.51	205	11	195	199	3	199	0.1639/ 0.2190
Large-BW 50k XGANDALF	3.5	11	51.77	39.52	12.26	181	10	161	204	1	204	0.1803/ 0.2440
Large-BW 135k pinkIndexer	2.05	9	57.07	39.00	18.07	200	7	168	199	3	199	0.2065/ 0.2070
Large-BW 102k	2.05	5	60.75	40.91	19.84	200	7	191	201	2	201	0.2064/

pinkIndexer												0.2140
Large-BW 30k pinkIndexer	3.2	9	46.68	37.03	9.66	165	10	121	200*	1	167	0.2700/ 0.3000

*Phenix *AutoBuild* was used to complete the initial model obtained from *autoSHARP*.

Table S2 Data quality indicators from CrystFEL of the SASE thaumatin data set with 102,277 indexed images processed with XGANDALF calculated up to resolution of 2.0 Å.

Resolution shell	Min resolution [Å]	Max resolution [Å]	Number of reflections	Redundancy	Rsplit [%]	CC 1/2	CC*	CC ano	[1] CC anoref	SNR
1	24.81	4.92	2280	687	3.8	0.997	0.999	0.353	0.587	25.68
2	4.92	3.91	2271	420	3.1	0.998	0.999	0.461	0.784	32.26
3	3.91	3.42	2262	382	3.7	0.959	0.990	0.055	0.676	29.55
4	3.42	3.11	2275	336	3.8	0.997	0.999	0.277	0.614	24.91
5	3.11	2.88	2256	252	4.9	0.995	0.999	0.156	0.597	19.04
6	2.88	2.71	2280	204	5.9	0.994	0.998	0.280	0.452	15.28
7	2.71	2.58	2259	172	7.1	0.992	0.998	0.116	0.465	13.24
8	2.58	2.47	2249	142	7.8	0.989	0.997	0.189	0.450	11.99
9	2.47	2.37	2259	116	8.2	0.988	0.997	0.179	0.375	11.44
10	2.37	2.29	2266	85	9.6	0.985	0.996	0.074	0.401	9.81
11	2.29	2.22	2261	72	10.6	0.979	0.995	0.137	0.340	8.91
12	2.22	2.15	2258	59	11.7	0.976	0.994	0.070	0.342	7.88
13	2.15	2.10	2276	42	13.6	0.966	0.991	0.117	0.284	6.73
14	2.10	2.05	2263	22	19.3	0.938	0.984	0.005	0.241	5.17
15	2.05	2.00	2261	11	27.3	0.869	0.964	0.145	0.263	4.42

1) CCanoref is the correlation coefficient between the measured (|ΔFobs|) and calculated (|ΔFcalc|) anomalous difference structure-factor amplitudes. The |Fcalc| were calculated from the thaumatin model refined against the corresponding data set with theoretical anomalous scattering contributions of S atoms incorporated into the refinement.

Table S3 Data quality indicators from CrystFEL of the large-BW thaumatin data set with 130,621 indexed images processed with XGANDALF calculated up to resolution of 2.2 Å.

Resolution shell	Min resolution [Å]	Max resolution [Å]	Number of reflections	Redundancy	Rsplit [%]	CC 1/2	CC*	CC ano	[1)] CC anoref	SNR
1	24.81	5.41	1713	1894	2.0	0.999	1.000	0.580	0.836	50.77
2	5.41	4.30	1720	1147	1.8	0.999	1.000	0.639	0.932	49.73
3	4.30	3.76	1693	1049	2.4	0.995	0.999	0.261	0.831	40.58
4	3.76	3.42	1703	948	2.8	0.999	1.000	0.280	0.790	31.80
5	3.42	3.17	1708	832	3.9	0.997	0.999	0.209	0.654	23.79
6	3.17	2.99	1693	654	5.2	0.995	0.999	0.230	0.599	17.78
7	2.99	2.84	1716	436	7.8	0.989	0.997	0.089	0.489	12.16
8	2.84	2.71	1698	319	9.6	0.985	0.996	0.035	0.459	9.71
9	2.71	2.61	1692	223	12.2	0.982	0.995	0.059	0.386	7.55
10	2.61	2.52	1682	153	15.0	0.961	0.990	0.104	0.296	6.25
11	2.52	2.44	1692	111	19.4	0.941	0.985	-0.002	0.215	4.89
12	2.44	2.37	1726	75	24.5	0.903	0.974	0.004	0.270	3.87
13	2.37	2.31	1707	48	36.3	0.847	0.958	0.046	0.115	2.56
14	2.31	2.25	1658	34	51.0	0.704	0.909	0.067	0.136	1.93
15	2.25	2.20	1738	24	73.3	0.594	0.863	0.022	0.046	1.38

Table S4 Overall data quality indicators from CrystFEL of the large-BW thaumatin data set with 130,621 indexed images processed with XGANDALF calculated up to resolution of 2.2 Å.

Rsplit [%]	3.37
CC ½	0.999
CC*	0.999
CC ano	0.476
SNR	17.89

Table S5 Data quality indicators from CrystFEL of the large-BW thaumatin data set with 102,000 indexed images processed with XGANDALF calculated up to resolution of 2.2 Å.

Resolution shell	Min resolution [Å]	Max resolution [Å]	Number of reflections	Redundancy	Rsplit [%]	CC 1/2	CC*	CC ano	[1)] CC anoref	SNR
1	24.81	5.41	1713	1495	2.2	0.999	1.000	0.586	0.833	45.52
2	5.41	4.30	1720	905	2.1	0.999	1.000	0.603	0.912	44.59
3	4.30	3.76	1693	827	2.6	0.996	0.999	0.283	0.813	36.49
4	3.76	3.42	1703	748	3.1	0.998	1.000	0.279	0.772	28.60
5	3.42	3.17	1708	656	4.4	0.996	0.999	0.107	0.665	21.32
6	3.17	2.99	1693	516	5.8	0.994	0.999	0.152	0.564	15.96
7	2.99	2.84	1716	345	9.0	0.985	0.996	0.065	0.457	10.82
8	2.84	2.71	1698	252	10.9	0.981	0.995	0.015	0.388	8.62
9	2.71	2.61	1692	175	13.9	0.974	0.993	0.101	0.348	6.68
10	2.61	2.52	1682	120	17.3	0.947	0.986	0.091	0.240	5.50
11	2.52	2.44	1692	87	22.1	0.923	0.980	0.014	0.200	4.29
12	2.44	2.37	1726	58	28.4	0.879	0.967	0.084	0.224	3.35
13	2.37	2.31	1707	38	43.3	0.789	0.939	0.012	0.100	2.24
14	2.31	2.25	1658	27	63.2	0.612	0.871	0.066	0.109	1.65
15	2.25	2.20	1738	19	89.6	0.501	0.817	-0.011	0.022	1.18

Table S6 Data quality indicators from CrystFEL of the large-BW thaumatin data set with 50,000 indexed images processed with *XGANDALF* calculated up to resolution of 2.2 Å.

Resolution shell	Min resolution [Å]	Max resolution [Å]	Number of reflections	Redundancy	Rsplit [%]	CC 1/2	CC*	CC ano	1) CC anoref	SNR
1	24.81	5.41	1713	731	3.3	0.997	0.999	0.295	0.728	31.61
2	5.41	4.30	1720	443	2.9	0.999	1.000	0.419	0.871	31.02
3	4.30	3.76	1693	404	3.6	0.998	0.999	0.319	0.777	25.50
4	3.76	3.42	1703	368	4.5	0.996	0.999	0.128	0.640	19.96
5	3.42	3.17	1708	322	6.2	0.993	0.998	0.119	0.563	14.84
6	3.17	2.99	1693	255	8.5	0.988	0.997	0.101	0.493	11.14
7	2.99	2.84	1716	170	12.8	0.971	0.993	0.168	0.355	7.56
8	2.84	2.71	1698	123	15.9	0.961	0.990	-0.026	0.273	6.01
9	2.71	2.61	1692	85	19.8	0.952	0.988	0.068	0.249	4.69
10	2.61	2.52	1682	58	25.5	0.895	0.972	0.000	0.162	3.82
11	2.52	2.44	1692	42	31.6	0.855	0.960	0.100	0.099	3.07
12	2.44	2.37	1726	29	41.6	0.763	0.930	0.037	0.166	2.39
13	2.37	2.31	1707	18	64.4	0.612	0.871	-0.037	0.037	1.71
14	2.31	2.25	1658	13	83.8	0.461	0.794	0.104	0.061	1.31
15	2.25	2.20	1738	9	104.2	0.424	0.772	0.133	0.060	0.94

Table S7 Data quality indicators from CrystFEL of the large-BW thaumatin data set with 135,482 indexed images processed with *pinkIndexer* calculated up to resolution of 2.05 Å.

Resolution shell	Min resolution [Å]	Max resolution [Å]	Number of reflections	Redundancy	Rsplit [%]	CC 1/2	CC*	CC ano	[1)] CC anoref	SNR
1	25.25	5.04	2132	2247	1.7	1.000	1.000	0.769	0.864	54.55
2	5.04	4.01	2103	2889	1.6	0.999	1.000	0.557	0.918	59.14
3	4.01	3.50	2103	3531	1.6	1.000	1.000	0.594	0.855	56.25
4	3.50	3.18	2088	3790	1.9	0.999	1.000	0.512	0.822	48.40
5	3.18	2.96	2123	3270	2.3	0.999	1.000	0.510	0.726	39.22
6	2.96	2.78	2088	2311	3.2	0.998	1.000	0.415	0.708	29.65
7	2.78	2.64	2127	1755	3.6	0.998	0.999	0.270	0.677	25.59
8	2.64	2.53	2083	1210	4.5	0.997	0.999	0.310	0.590	21.18
9	2.53	2.43	2082	876	5.1	0.995	0.999	0.201	0.586	18.39
10	2.43	2.35	2104	602	6.2	0.993	0.998	0.268	0.511	14.81
11	2.35	2.27	2107	401	9.5	0.985	0.996	0.126	0.485	9.90
12	2.27	2.21	2128	300	14.2	0.965	0.991	0.009	0.440	6.67
13	2.21	2.15	2077	203	26.7	0.913	0.977	-0.034	0.318	3.67
14	2.15	2.10	2106	107	45.4	0.801	0.943	0.015	0.249	2.15
15	2.10	2.05	2114	39	81.9	0.597	0.865	0.078	0.117	1.16

Table S8 Overall data quality indicators from CrystFEL of the large-BW thaumatin data set with 135,482 indexed images processed with *pinkIndexer* calculated up to resolution of 2.05 Å.

Rsplit [%]	2.10
CC ½	0.999
CC*	0.999
CC ano	0.746
SNR	26.06

Table S9 Data quality indicators from CrystFEL of the large-BW thaumatin data set with 102,000 indexed images processed with *pinkIndexer* calculated up to resolution of 2.05 Å.

Resolution shell	Min resolution [Å]	Max resolution [Å]	Number of reflections	Redundancy	Rsplit [%]	CC 1/2	CC*	CC ano	[1)]CC anoref	SNR
1	25.25	5.04	2132	1738	2.0	0.999	1.000	0.685	0.827	49.11
2	5.04	4.01	2103	2250	1.8	0.999	1.000	0.529	0.921	53.10
3	4.01	3.50	2103	2756	1.8	0.999	1.000	0.498	0.847	50.32
4	3.50	3.18	2088	2955	2.1	0.999	1.000	0.452	0.811	43.15
5	3.18	2.96	2123	2548	2.6	0.999	1.000	0.418	0.710	34.84
6	2.96	2.78	2088	1797	3.6	0.998	0.999	0.375	0.698	26.21
7	2.78	2.64	2127	1359	4.2	0.997	0.999	0.261	0.674	22.51
8	2.64	2.53	2083	931	5.1	0.996	0.999	0.284	0.595	18.68
9	2.53	2.43	2082	672	5.8	0.994	0.998	0.171	0.553	16.18
10	2.43	2.35	2104	462	7.4	0.990	0.997	0.227	0.456	12.86
11	2.35	2.27	2107	308	11.6	0.978	0.994	0.073	0.420	8.38
12	2.27	2.21	2128	232	17.6	0.952	0.988	0.060	0.395	5.43
13	2.21	2.15	2077	157	34.2	0.876	0.966	0.043	0.275	2.96
14	2.15	2.10	2106	83	57.0	0.741	0.923	-0.038	0.205	1.79
15	2.10	2.05	2114	30	100.6	0.426	0.773	-0.002	0.085	1.01

Table S10 Overall data quality indicators from CrystFEL of the large-BW thaumatin data set with 102,000 indexed images processed with *pinkIndexer* calculated up to resolution of 2.2 Å.

Rsplit [%]	2.20
CC ½	0.999
CC*	0.999
CC ano	0.672
SNR	28.14

Table S11 Data quality indicators from CrystFEL of the large-BW thaumatin data set with 102,000 indexed images processed with *pinkIndexer* calculated up to resolution of 2.2 Å.

Resolution shell	Min resolution [Å]	Max resolution [Å]	Number of reflections	Redundancy	Rsplit [%]	CC 1/2	CC*	CC ano	SNR
1	25.25	5.41	1713	1681	2.0	0.999	1.000	0.690	48.61
2	5.41	4.30	1721	2075	1.8	0.999	1.000	0.505	52.40
3	4.30	3.76	1693	2578	1.7	0.999	1.000	0.509	52.28
4	3.76	3.42	1700	2858	1.8	0.999	1.000	0.540	48.32
5	3.42	3.17	1707	2945	2.2	0.999	1.000	0.425	41.98
6	3.17	2.99	1697	2609	2.5	0.999	1.000	0.453	35.95
7	2.99	2.84	1716	1918	3.4	0.998	0.999	0.362	27.47
8	2.84	2.71	1696	1544	3.9	0.997	0.999	0.294	24.02
9	2.71	2.61	1694	1193	4.6	0.997	0.999	0.287	20.57
10	2.61	2.52	1682	870	5.1	0.995	0.999	0.287	18.56
11	2.52	2.44	1692	677	5.9	0.994	0.998	0.157	16.18
12	2.44	2.37	1726	500	6.8	0.992	0.998	0.235	13.74
13	2.37	2.31	1707	351	9.8	0.983	0.996	0.116	9.90
14	2.31	2.25	1658	274	13.9	0.966	0.991	0.080	7.18
15	2.25	2.20	1738	214	20.3	0.942	0.985	0.014	4.66

Table S12 Data quality indicators from CrystFEL of the large-BW thaumatin data set with 30,000 indexed images processed with *pinkIndexer* calculated up to resolution of 2.05 Å.

Resolution shell	Min resolution [Å]	Max resolution [Å]	Number of reflections	Redundancy	Rsplit [%]	CC 1/2	CC*	CC ano	[1)] CC anoref	SNR
1	25.25	5.04	2132	501	3.8	0.996	0.999	0.528	0.692	27.94
2	5.04	4.01	2103	657	3.2	0.998	0.999	0.342	0.809	29.40
3	4.01	3.50	2103	811	3.3	0.998	1.000	0.298	0.656	27.71
4	3.50	3.18	2088	875	3.9	0.997	0.999	0.210	0.671	23.55
5	3.18	2.96	2123	763	5.1	0.996	0.999	0.103	0.479	18.99
6	2.96	2.78	2088	535	6.7	0.993	0.998	0.205	0.454	14.18
7	2.78	2.64	2127	398	8.2	0.988	0.997	0.022	0.440	12.23
8	2.64	2.53	2083	268	9.3	0.986	0.997	0.137	0.414	10.15
9	2.53	2.43	2082	191	11.0	0.978	0.994	0.096	0.389	8.79
10	2.43	2.35	2104	130	14.4	0.962	0.990	0.052	0.285	7.04
11	2.35	2.27	2107	84	20.7	0.933	0.982	0.077	0.290	4.76
12	2.27	2.21	2128	61	30.7	0.849	0.958	0.046	0.230	3.41
13	2.21	2.15	2077	40	53.2	0.664	0.893	-0.059	0.160	2.00
14	2.15	2.10	2106	21	93.5	0.436	0.779	-0.067	0.100	1.18
15	2.10	2.05	2114	8	151.5	0.218	0.599	0.078	0.022	1.02

Table S13 Overall data quality indicators from CrystFEL of the large-BW thaumatin data set with 30,000 indexed images processed with *pinkIndexer* calculated up to resolution of 2.2 Å.

Rsplit [%]	4.15
CC ½	0.996
CC*	0.999
CC ano	0.511
SNR	15.54

Table S14 Data quality indicators from CrystFEL of the large-BW thaumatin data set with 30,000 indexed images processed with *pinkIndexer* calculated up to resolution of 2.2 Å.

Resolution shell	Min resolution [Å]	Max resolution [Å]	Number of reflections	Redundancy	Rsplit [%]	CC 1/2	CC*	CC ano	SNR
1	25.25	5.41	1713	482	3.8	0.996	0.999	0.531	27.74
2	5.41	4.30	1721	606	3.1	0.998	1.000	0.343	29.15
3	4.30	3.76	1693	756	3.2	0.998	0.999	0.297	28.86
4	3.76	3.42	1700	842	3.4	0.998	0.999	0.288	26.52
5	3.42	3.17	1707	873	4.0	0.997	0.999	0.189	22.91
6	3.17	2.99	1697	782	4.9	0.996	0.999	0.143	19.61
7	2.99	2.84	1716	573	6.5	0.993	0.998	0.162	14.87
8	2.84	2.71	1696	455	7.5	0.991	0.998	0.064	13.02
9	2.71	2.61	1694	348	8.7	0.988	0.997	0.083	11.17
10	2.61	2.52	1682	250	9.3	0.985	0.996	0.170	10.09
11	2.52	2.44	1692	192	11.1	0.978	0.994	0.083	8.80
12	2.44	2.37	1726	141	13.3	0.968	0.992	0.050	7.50
13	2.37	2.31	1707	97	18.2	0.945	0.986	0.061	5.50
14	2.31	2.25	1658	74	23.8	0.904	0.974	0.066	4.25
15	2.25	2.20	1738	56	35.0	0.817	0.948	0.023	2.99

Table S15 Data quality indicators from CrystFEL of the SASE thaumatin data set processed with *XGANDALF* calculated up to resolution of 2.2 Å.

Resolution shell	Min resolution [Å]	Max resolution [Å]	Number of reflections	Redundancy	Rsplit [%]	CC 1/2	CC*	CC ano	SNR
1	24.81	5.41	1713	755	3.9	0.996	0.999	0.363	24.89
2	5.41	4.30	1720	442	3.2	0.998	0.999	0.460	30.32
3	4.30	3.76	1693	409	3.1	0.998	0.999	0.360	32.23
4	3.76	3.42	1703	377	3.9	0.940	0.984	0.043	29.08
5	3.42	3.17	1708	343	3.7	0.997	0.999	0.280	25.57
6	3.17	2.99	1693	288	4.4	0.996	0.999	0.179	21.70
7	2.99	2.84	1716	223	5.5	0.994	0.999	0.224	16.77
8	2.84	2.71	1698	201	6.1	0.994	0.998	0.263	14.93
9	2.71	2.61	1692	175	7.1	0.993	0.998	0.142	13.24
10	2.61	2.52	1682	152	7.4	0.990	0.997	0.139	12.67
11	2.52	2.44	1692	133	7.9	0.989	0.997	0.199	11.71
12	2.44	2.37	1726	111	8.4	0.987	0.997	0.157	11.32
13	2.37	2.31	1707	87	9.5	0.985	0.996	0.090	9.80
14	2.31	2.25	1658	77	10.2	0.981	0.995	0.144	9.25
15	2.25	2.20	1738	67	11.0	0.978	0.994	0.089	8.51

Figure S8 Simulated snapshot diffraction patterns from a randomly oriented crystal of the 5-HT_{2B} receptor (PDB 4NC3) with mosaicity of 0 degrees with X-ray bandwidth of 3.5 % and photon energy of 4.5 keV and experimental geometry similar to the SFX setup at the Alvra end station at SwissFEL with the JUNGFRAU 16M detector. The resolution at the edge of the image is 2.6 Å.

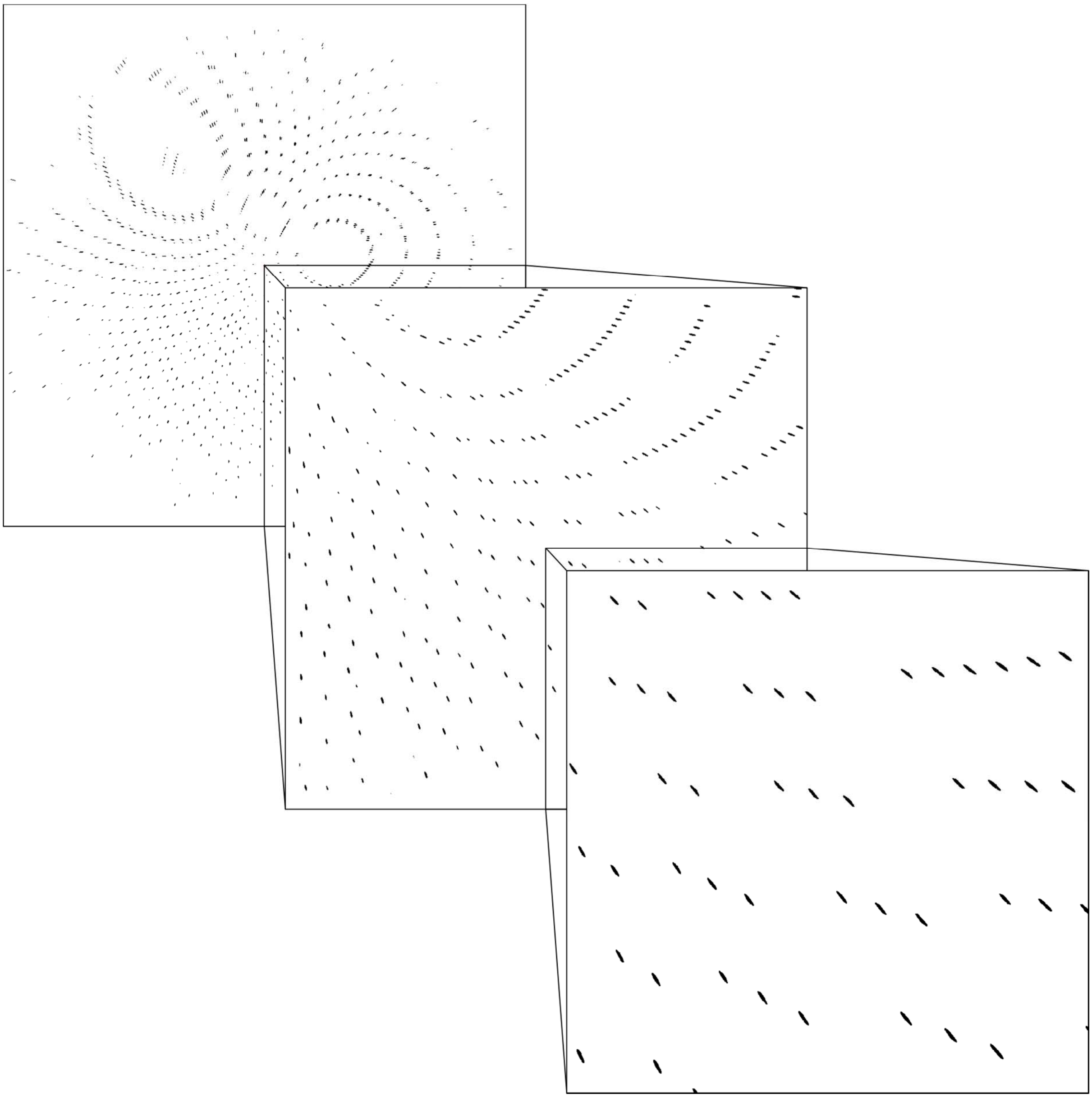

Figure S9 Simulated snapshot diffraction patterns from a randomly oriented crystal of the 5-HT_{2B} receptor (PDB 4NC3) with mosaicity of 0.1 degrees with X-ray bandwidth of 3.5 % and photon energy of 4.5 keV and experimental geometry similar to the SFX setup at the Alvra end station at SwissFEL with the JUNGFRAU 16M detector. The resolution at the edge of the image is 2.6 Å.

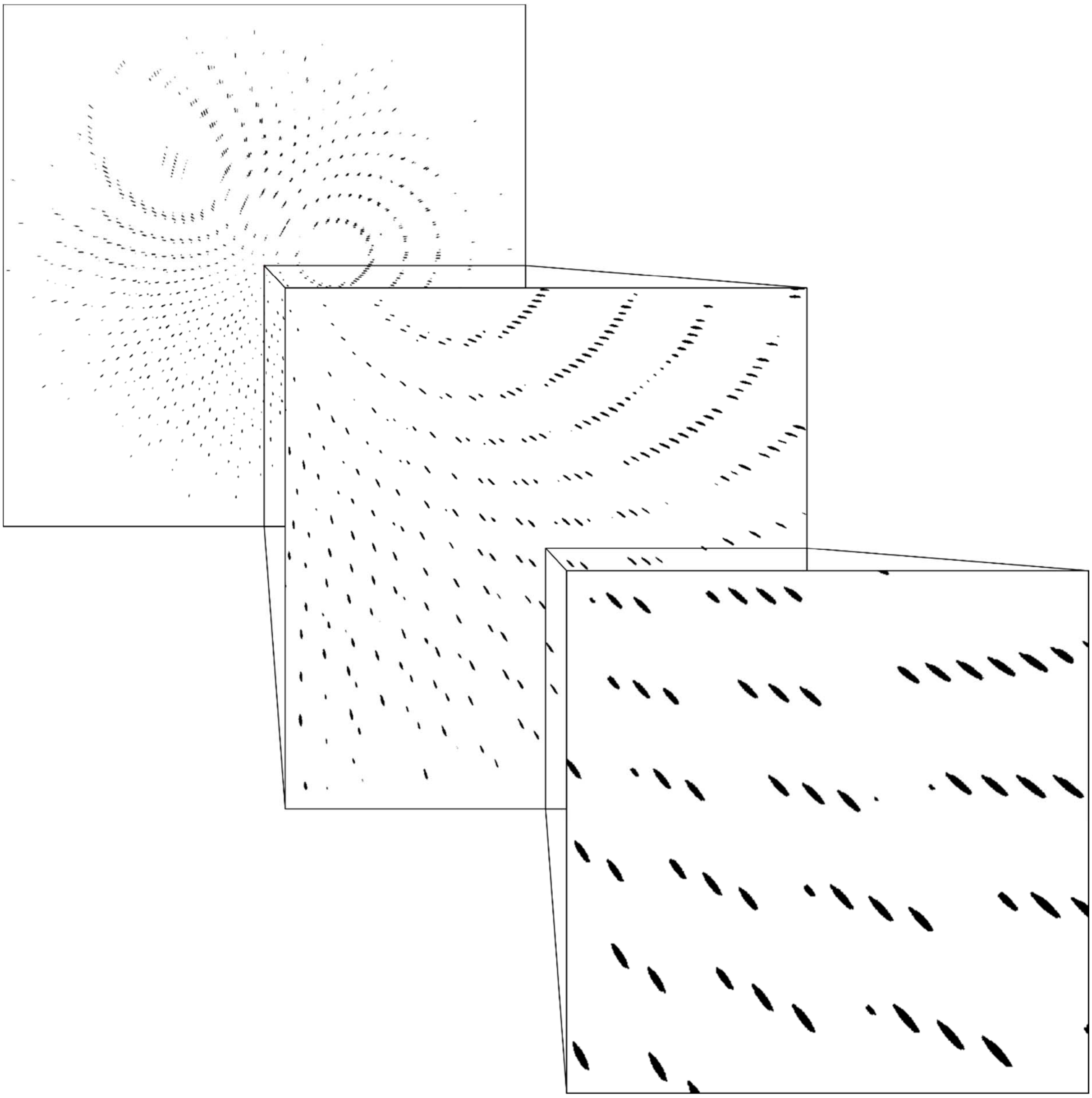

Figure S10 Simulated snapshot diffraction patterns from a randomly oriented crystal of the 5-HT_{2B} receptor (PDB 4NC3) with mosaicity of 0.3 degrees with X-ray bandwidth of 3.5 % and photon energy of 4.5 keV and experimental geometry similar to the SFX setup at the Alvra end station at SwissFEL with the JUNGFRAU 16M detector. The resolution at the edge of the image is 2.6 Å.

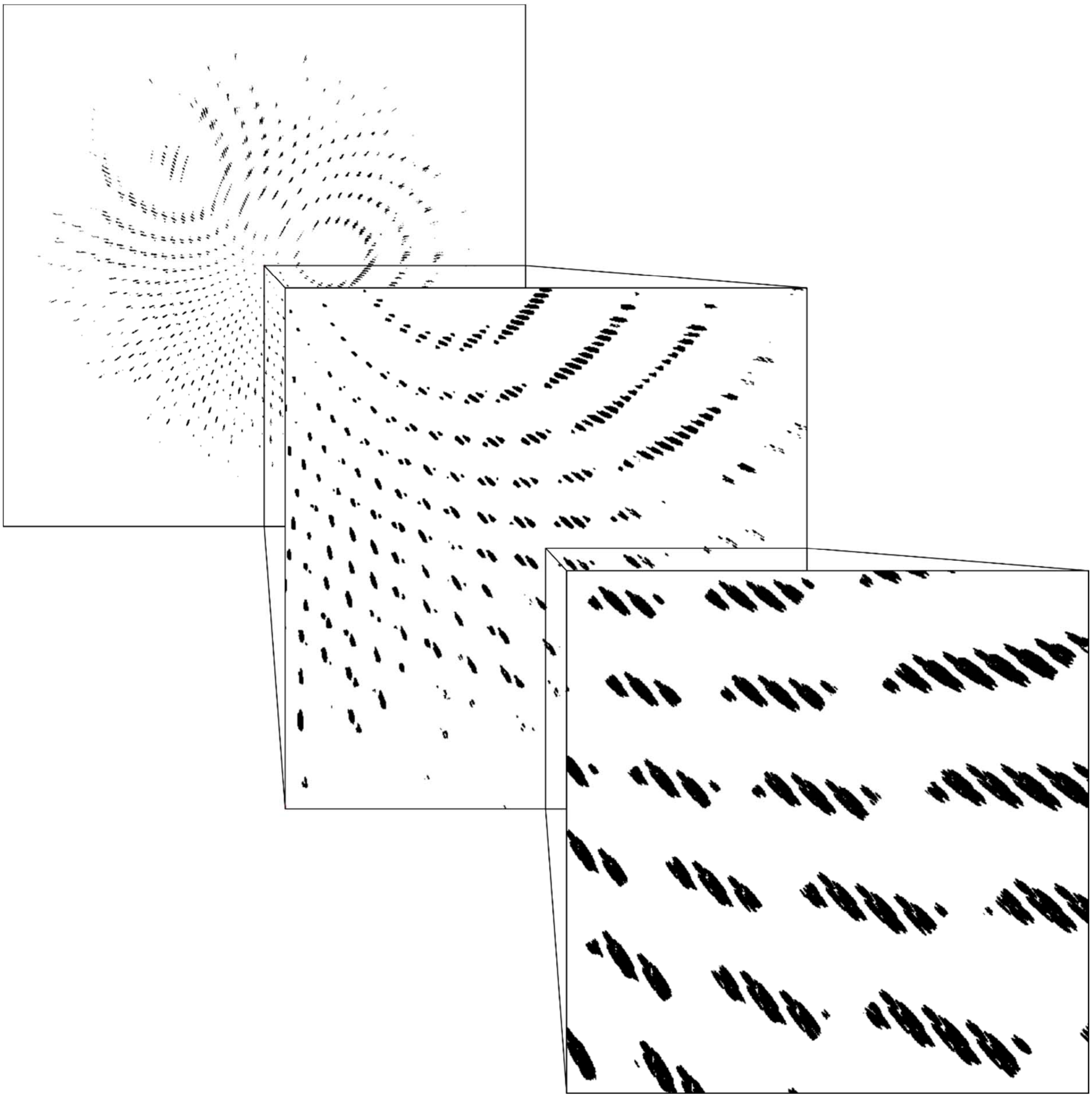

Figure S11 Simulated snapshot diffraction patterns from a randomly oriented crystal of the 5-HT$_{2B}$ receptor (PDB 4NC3) with mosaicity of 0 degrees with X-ray bandwidth of 3.0 % and photon energy of 4.5 keV and experimental geometry similar to the SFX setup at the Alvra end station at SwissFEL with the JUNGFRAU 16M detector. The resolution at the edge of the image is 2.6 Å.

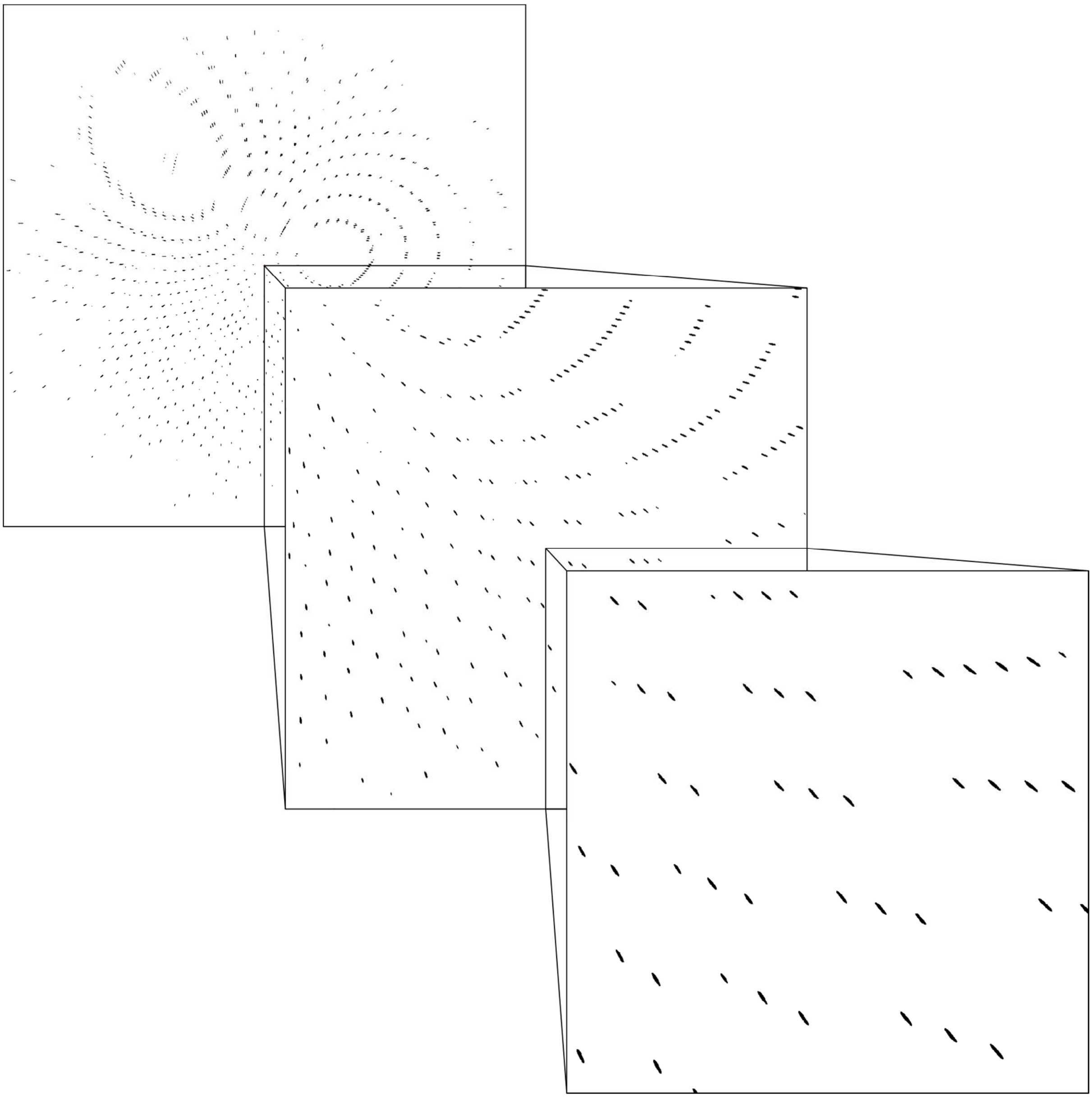

Figure S12 Simulated snapshot diffraction patterns from a randomly oriented crystal of the 5-HT_{2B} receptor (PDB 4NC3) with mosaicity of 0.1 degrees with X-ray bandwidth of 3.0 % and photon energy of 4.5 keV and experimental geometry similar to the SFX setup at the Alvra end station at SwissFEL with the JUNGFRAU 16M detector. The resolution at the edge of the image is 2.6 Å.

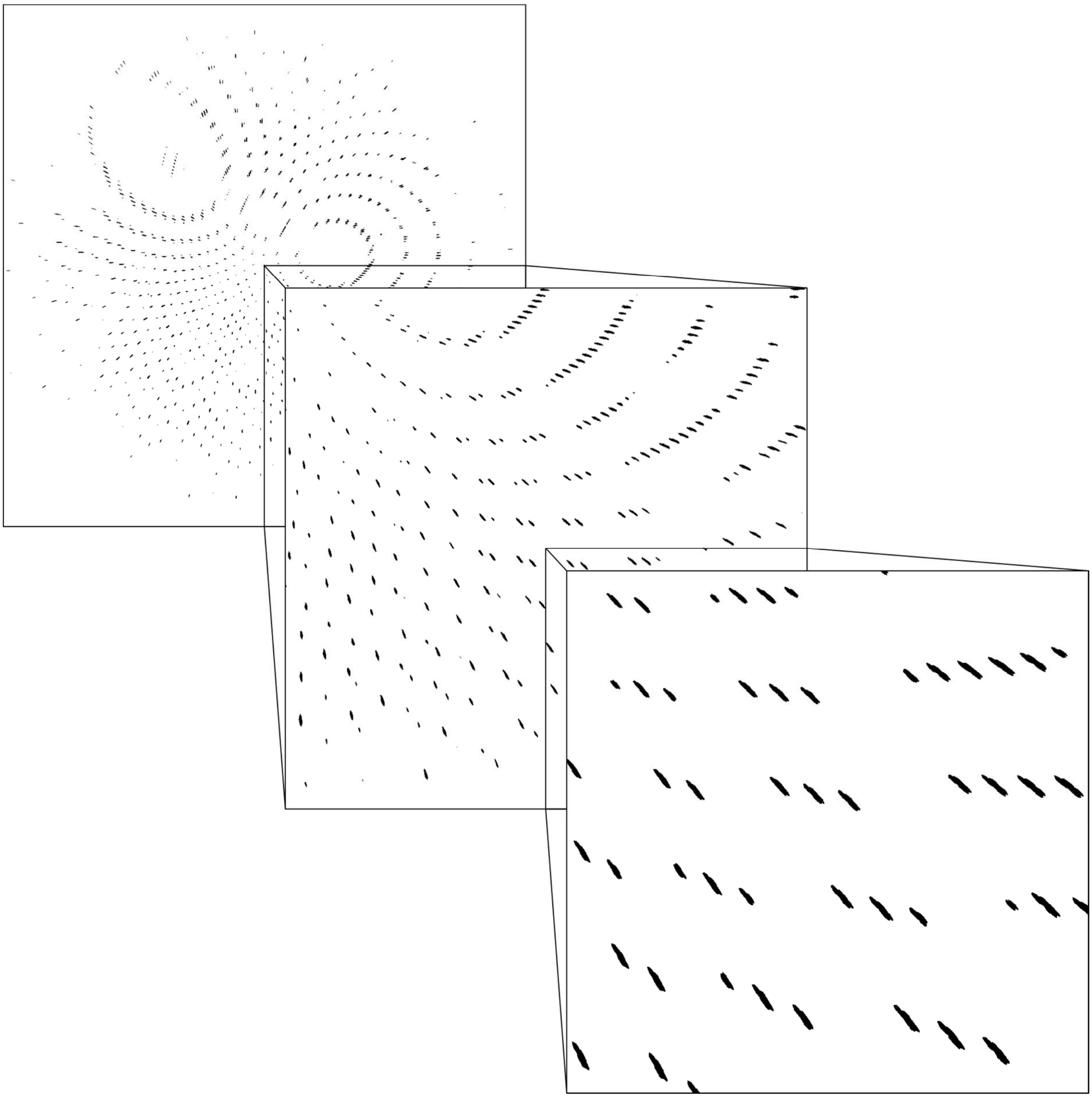

Figure S13 Simulated snapshot diffraction patterns from a randomly oriented crystal of the 5-HT_{2B} receptor (PDB 4NC3) with mosaicity of 0.2 degrees with X-ray bandwidth of 3.0 % and photon energy of 4.5 keV and experimental geometry similar to the SFX setup at the Alvra end station at SwissFEL with the JUNGFRAU 16M detector. The resolution at the edge of the image is 2.6 Å.

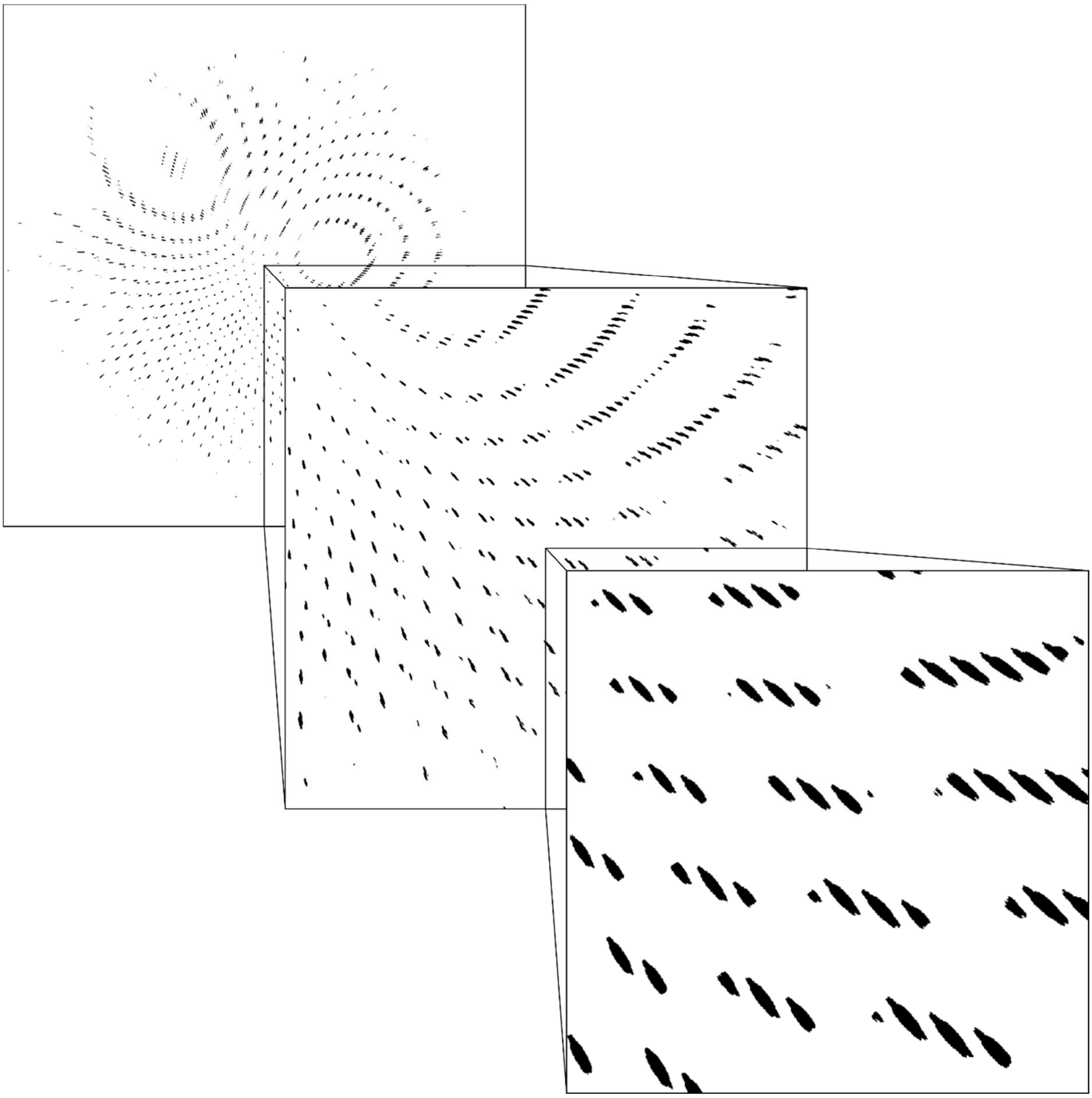

Figure S14 Simulated snapshot diffraction patterns from a randomly oriented crystal of the 5-HT_{2B} receptor (PDB 4NC3) with mosaicity of 0.3 degrees with X-ray bandwidth of 3.0 % and photon energy of 4.5 keV and experimental geometry similar to the SFX setup at the Alvra end station at SwissFEL with the JUNGFRAU 16M detector. The resolution at the edge of the image is 2.6 Å.

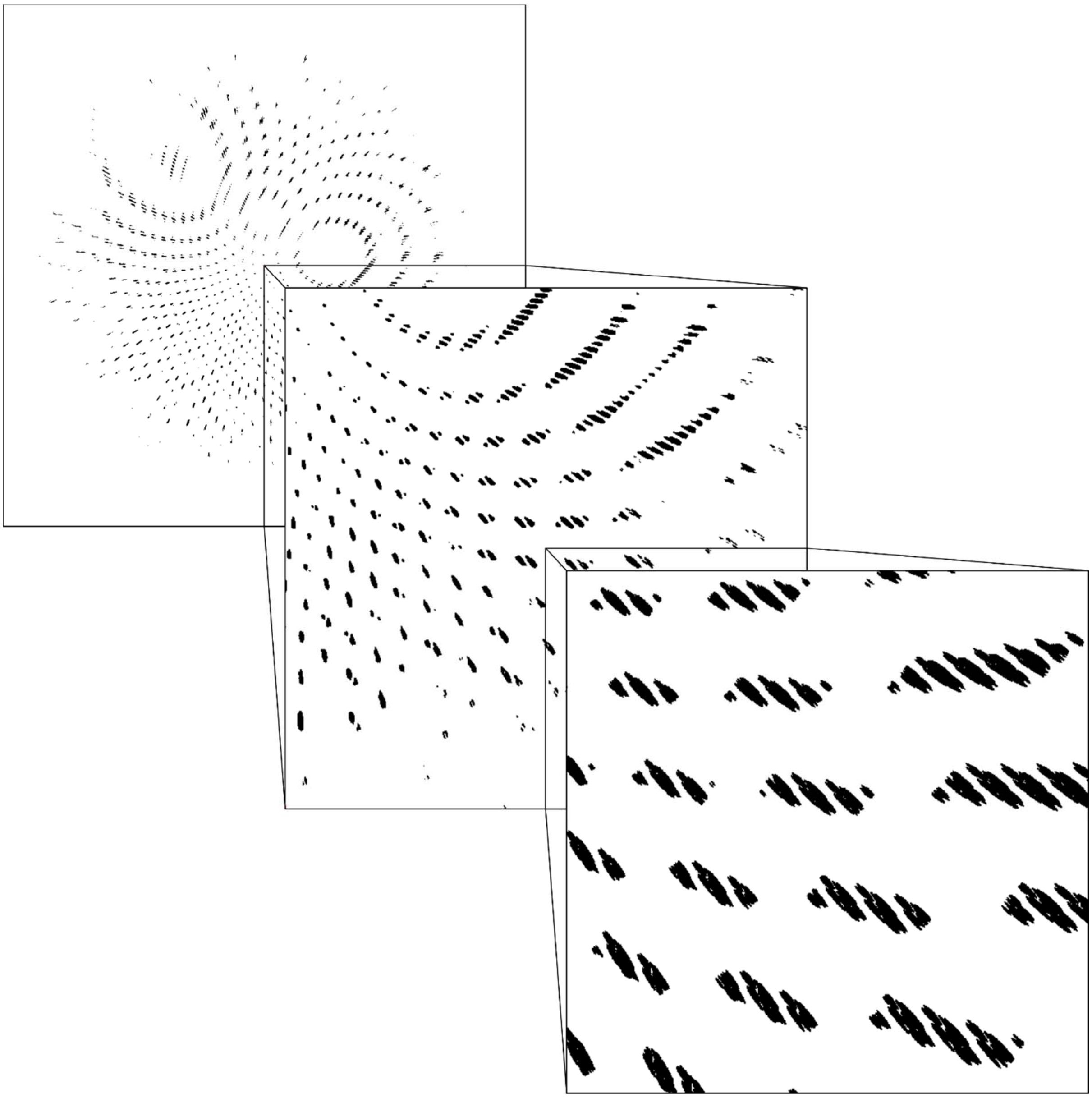

Figure S15 Simulated snapshot diffraction patterns from a randomly oriented crystal of the 5-HT$_{2B}$ receptor (PDB 4NC3) demonstrating the influence of various nanoBragg parameters on the simulated Bragg spot shapes and granularity at high-resolution.

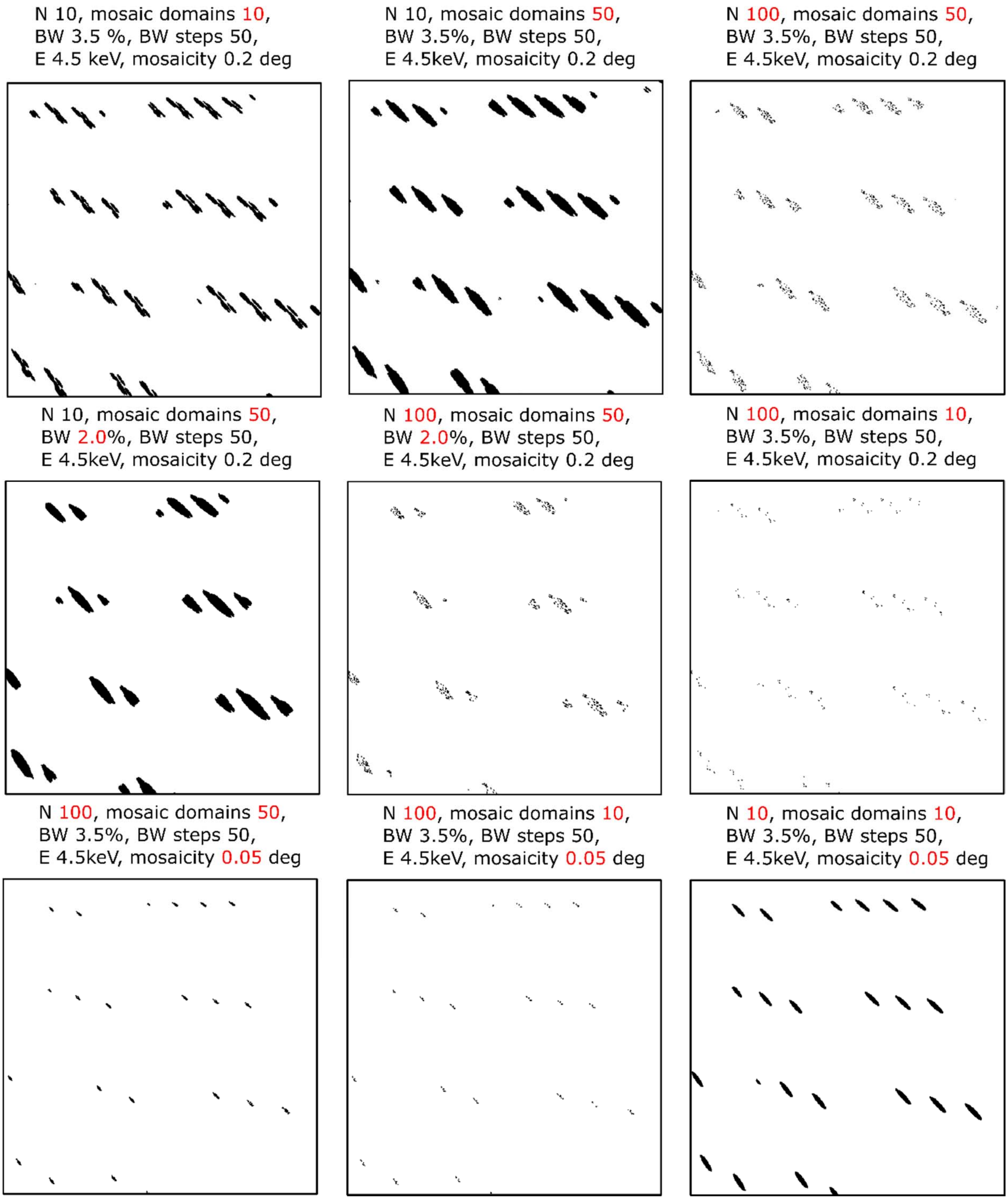

S1. Supplementary diffraction pattern gallery

a) Typical diffraction pattern from a single thaumatin microcrystal obtained with large-BW XFEL pulse ($\Delta E/E \sim 2.2\%$) from SwissFEL recorded by JUNGFRAU 16M detector at Alvra end-station. Visible are: diffuse background scattering from He/air and LCP/water ring, Bragg spots, gaps between the 1M modules and shadows from the beamstop and its holder, high-viscosity injector and catcher. One of the 1M modules on the bottom of the image was not functioning.

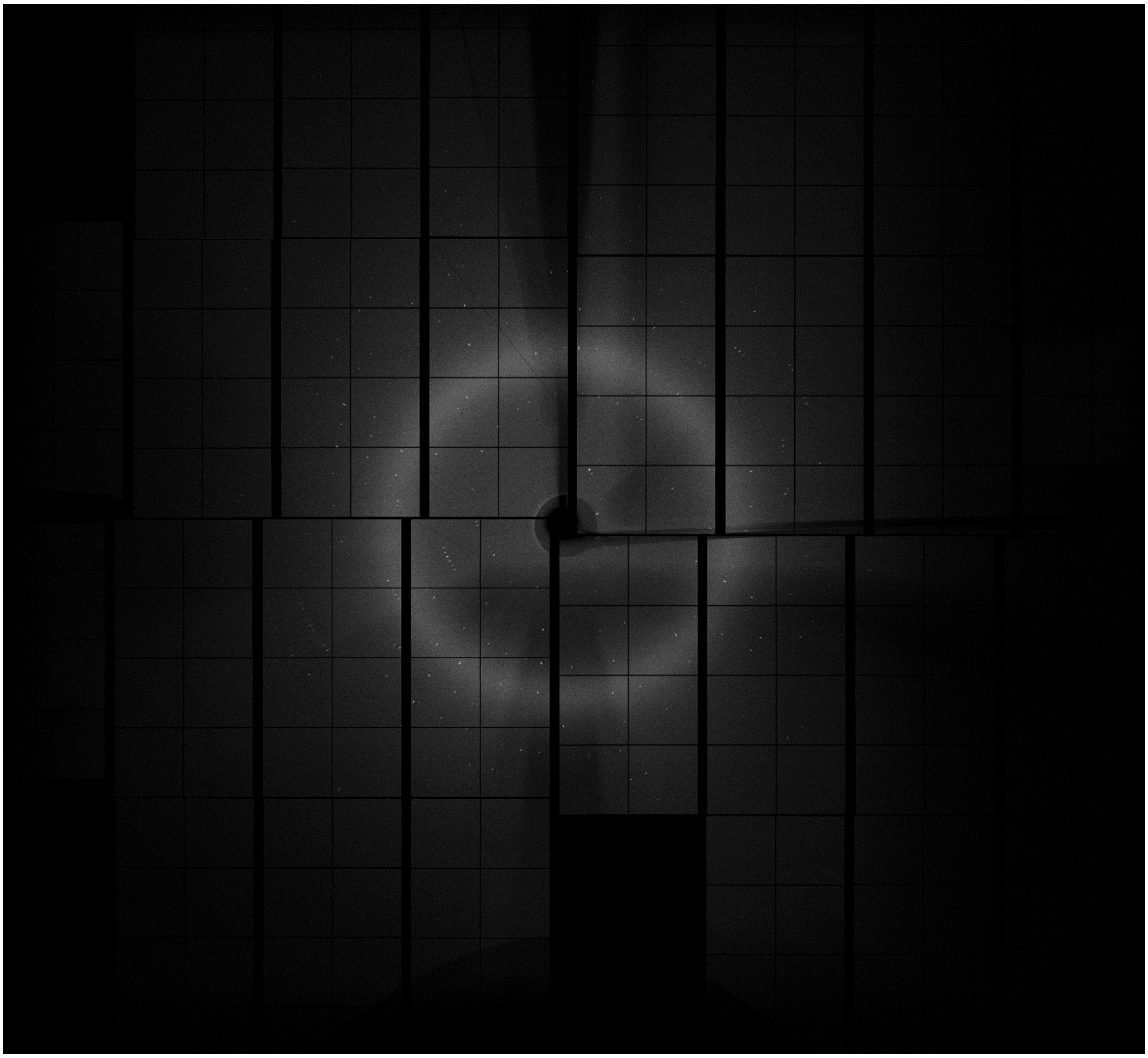

b) Zoom-in to the upper and lower half of the diffraction pattern from a).

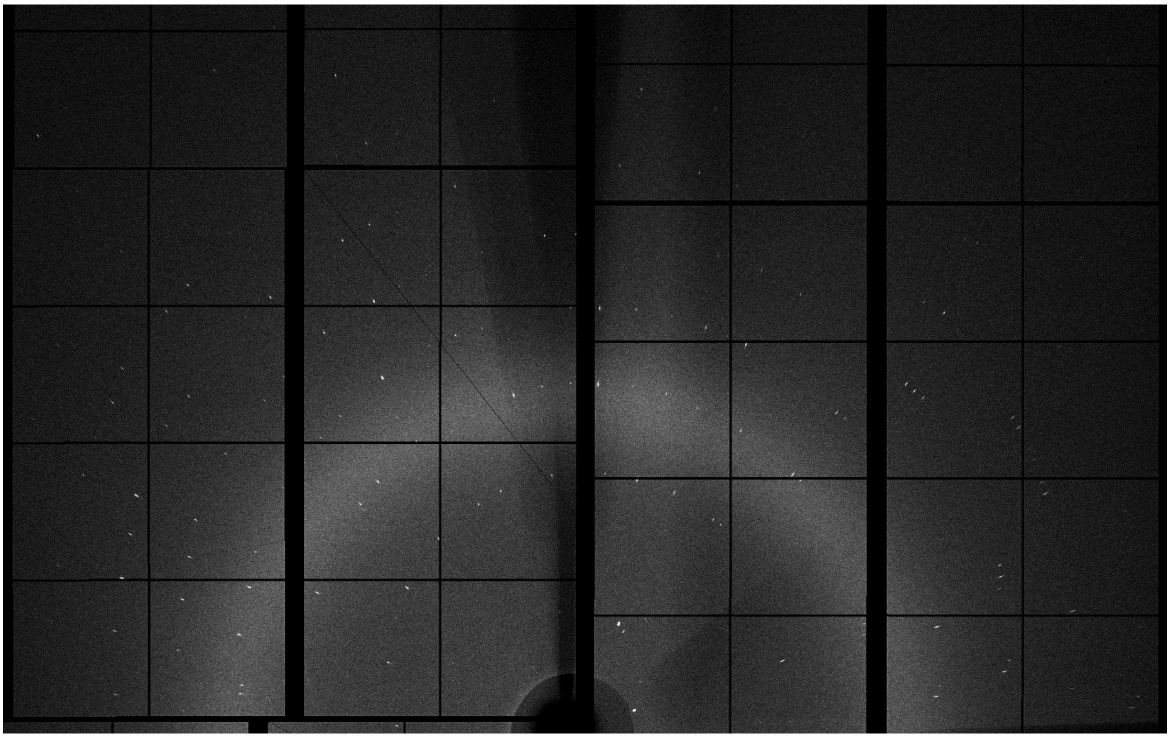

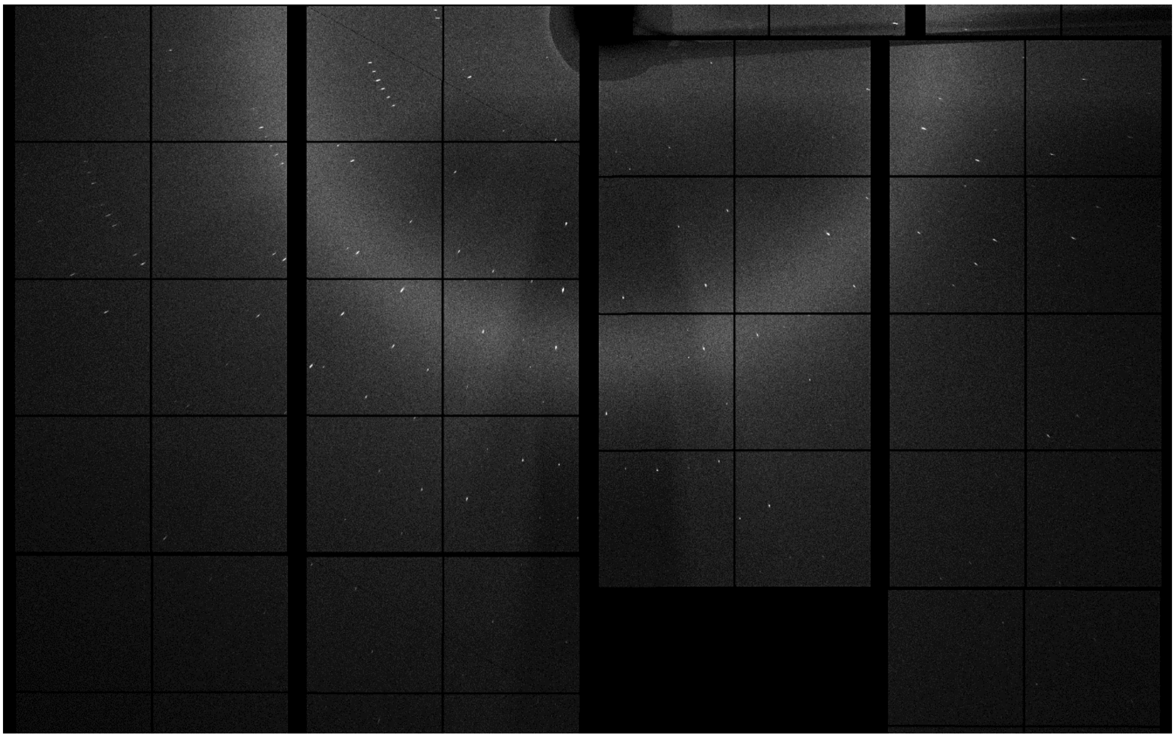

c) Zoom-in to the upper and lower left corners of the diffraction patterns from a).

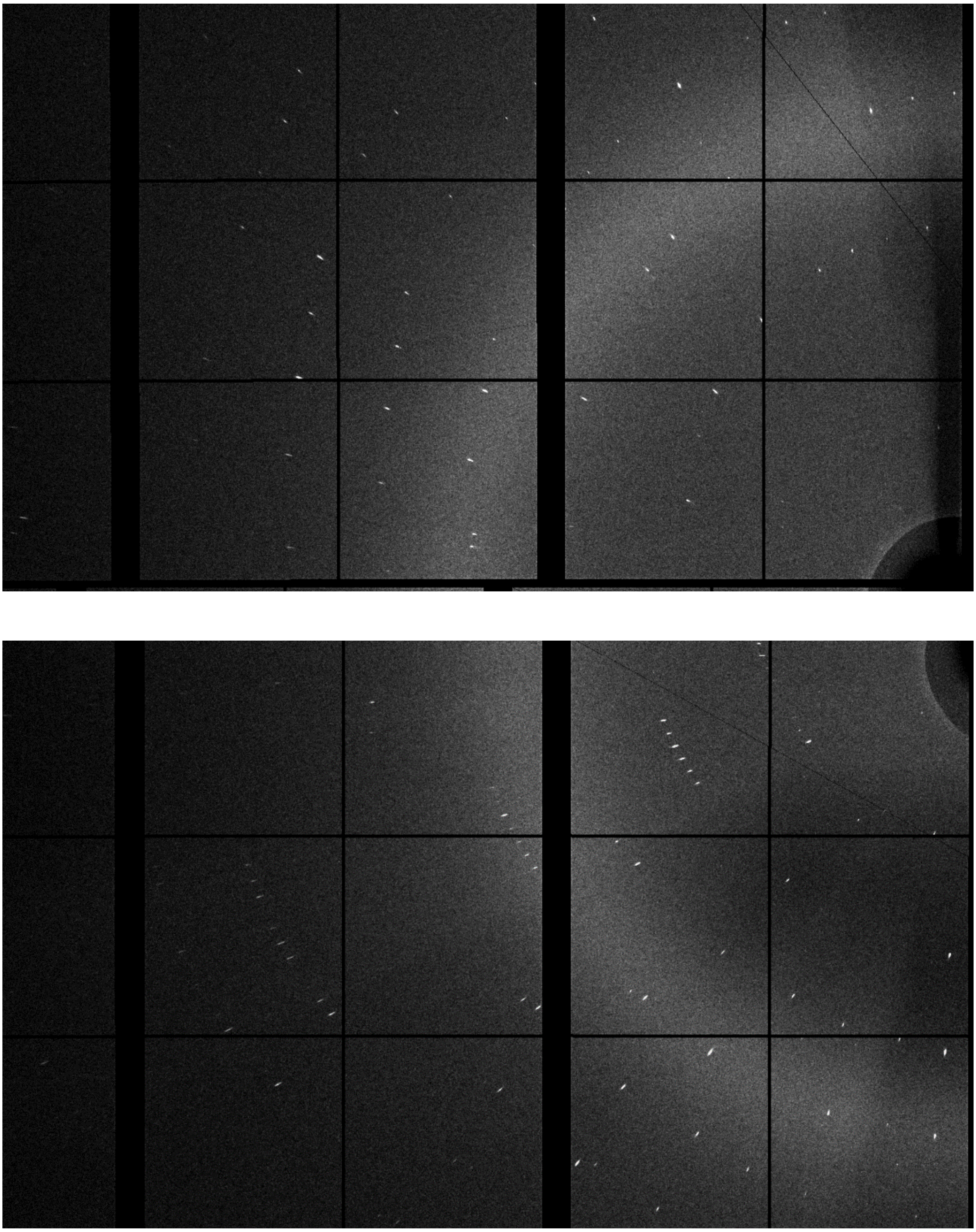

d) Another typical diffraction pattern from a single thaumatin microcrystal obtained with large-BW XFEL pulse (ΔE/E ~ 2.2%) from SwissFEL recorded by JUNGFRAU 16M detector at Alvra end-station.

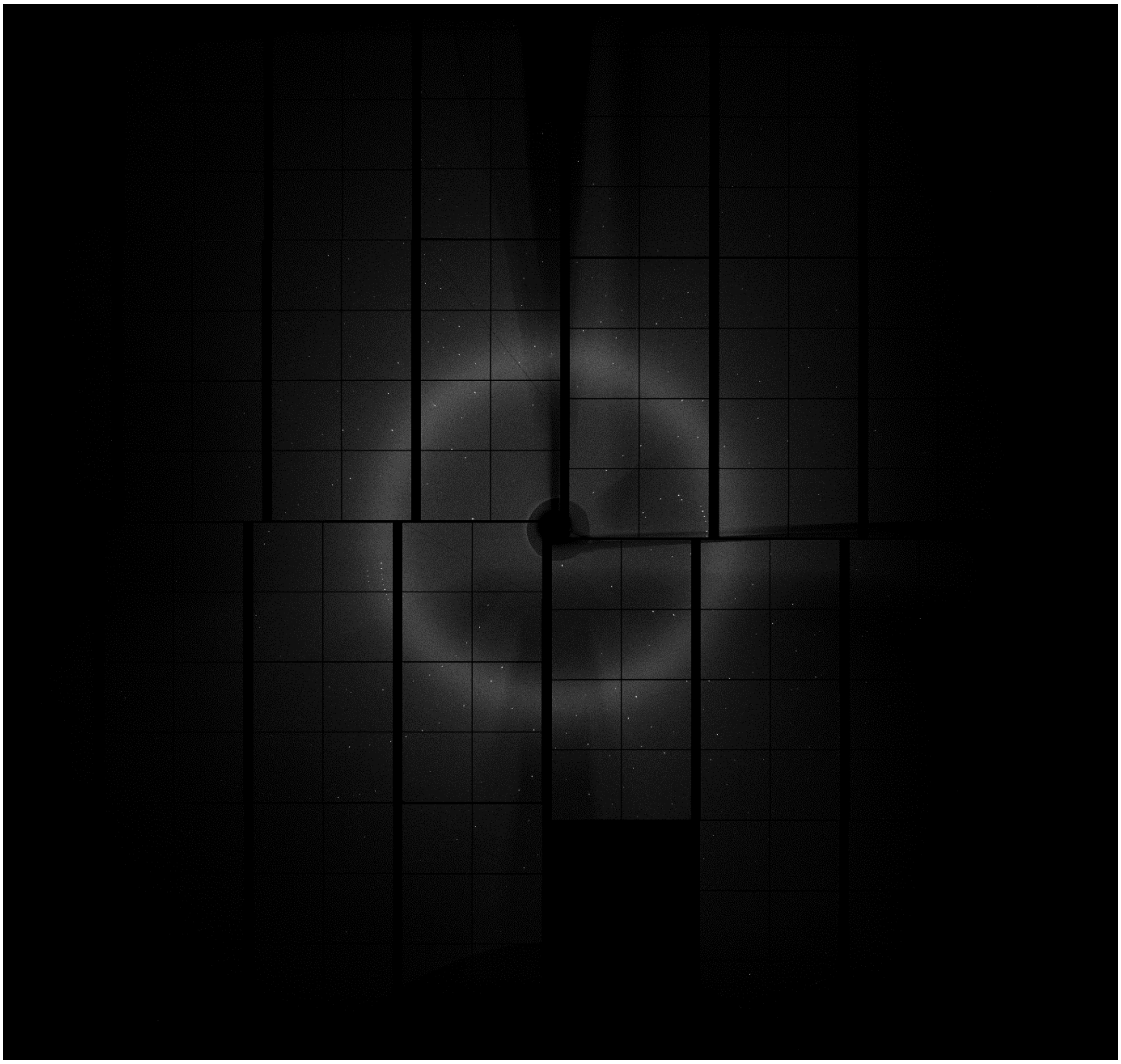

e) Zoom-in into the central and bottom right regions of the diffraction pattern from d).

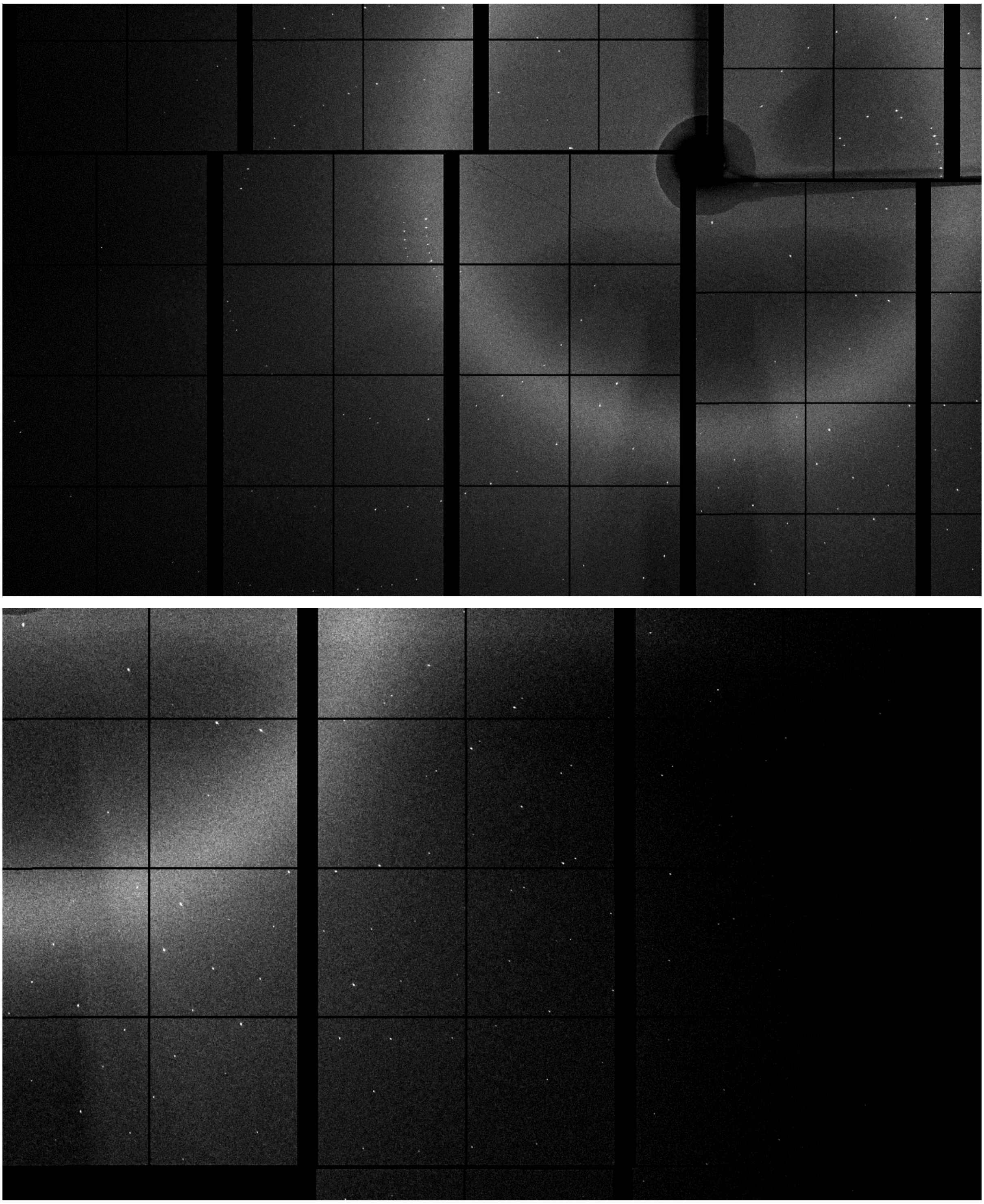

f) Another typical diffraction pattern from a single thaumatin microcrystal obtained with large-BW XFEL pulse (ΔE/E ~ 2.2%) from SwissFEL recorded by JUNGFRAU 16M detector at Alvra end-station.

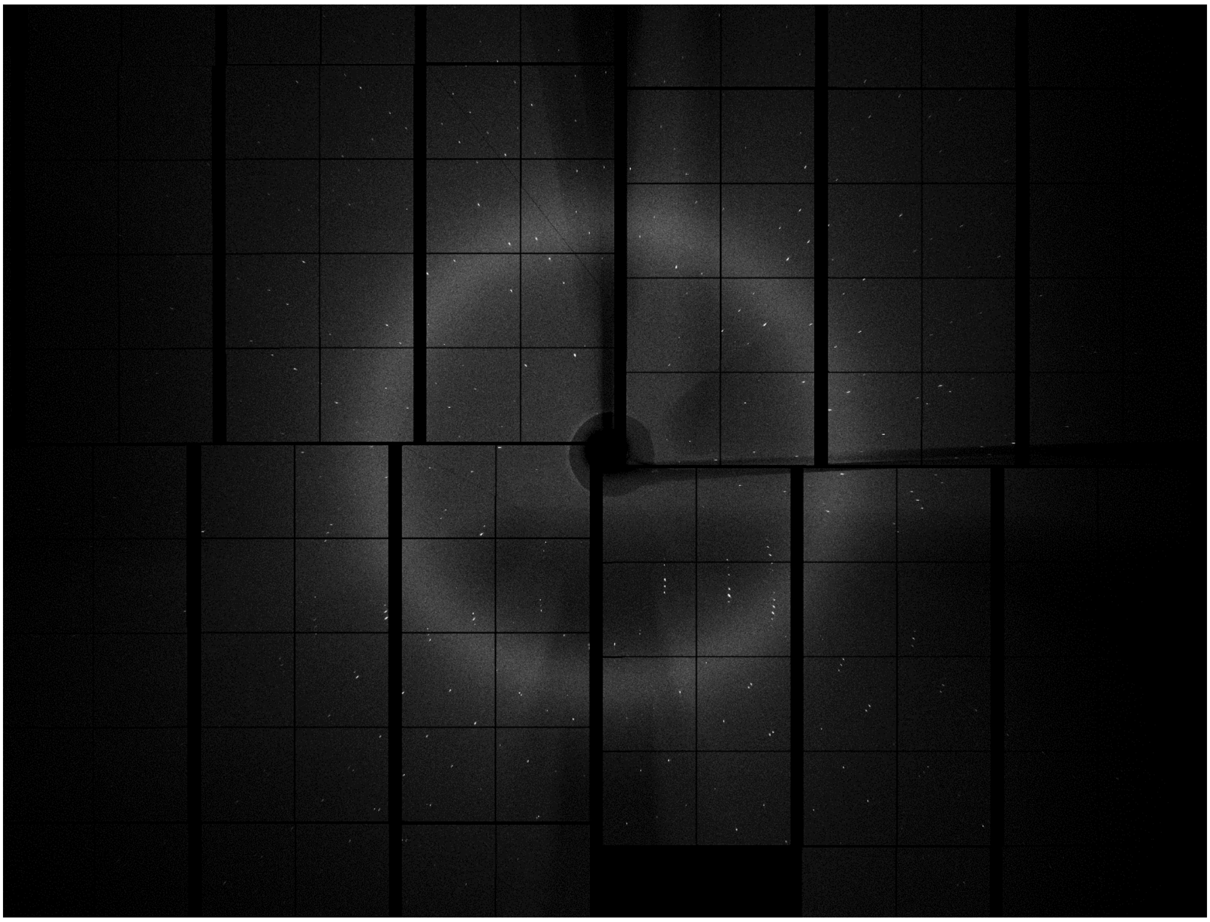

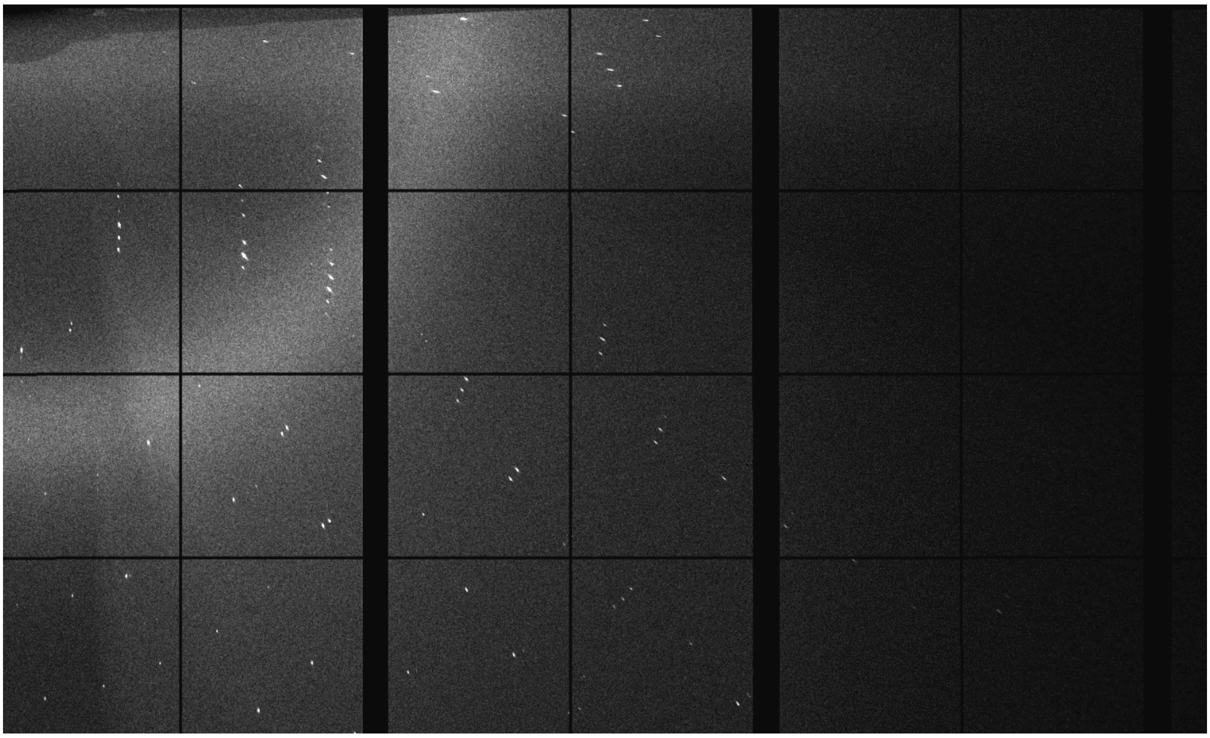

g) Typical diffraction pattern from a single thaumatin microcrystal obtained with nominal SASE XFEL pulse (ΔE/E ~ 0.17%) from SwissFEL recorded by JUNGFRAU 16M detector at Alvra end-station.

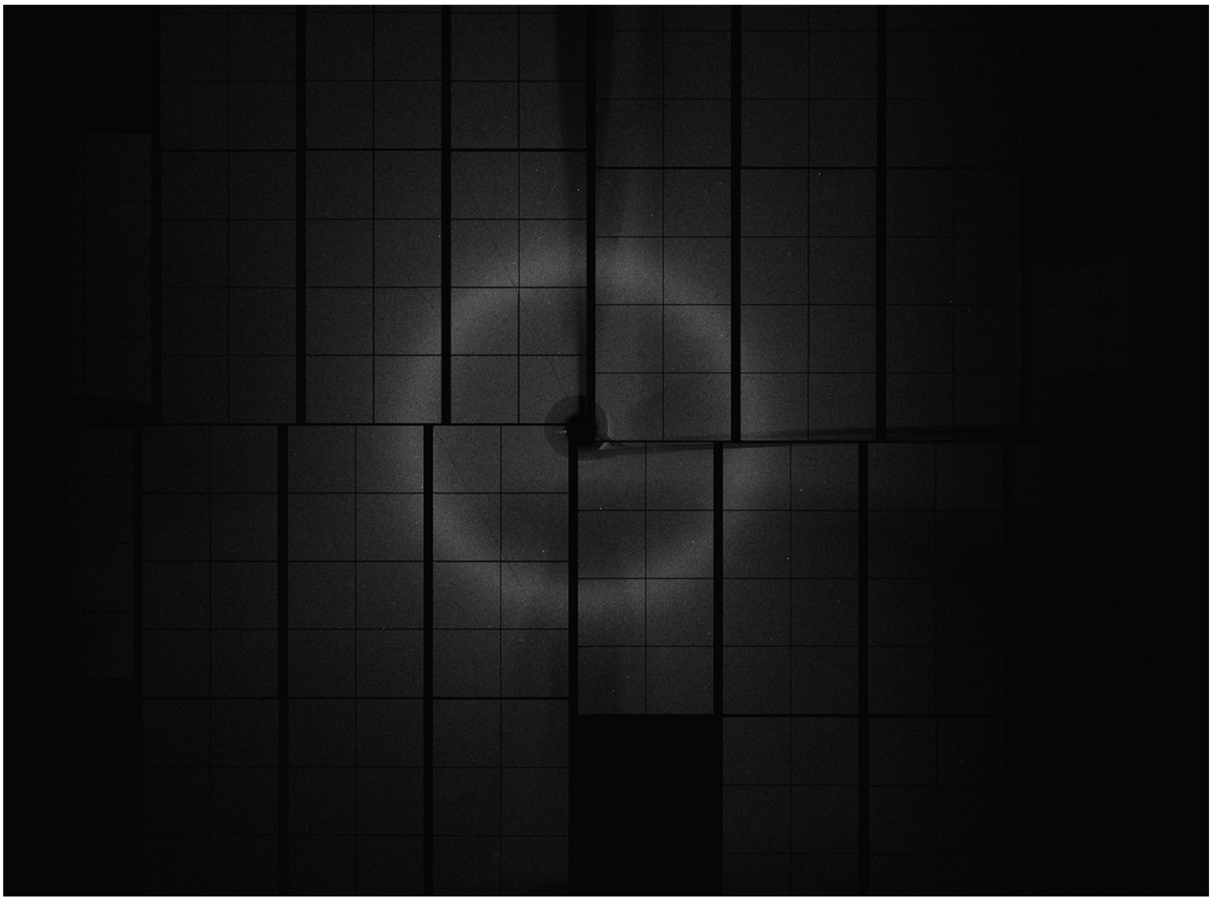

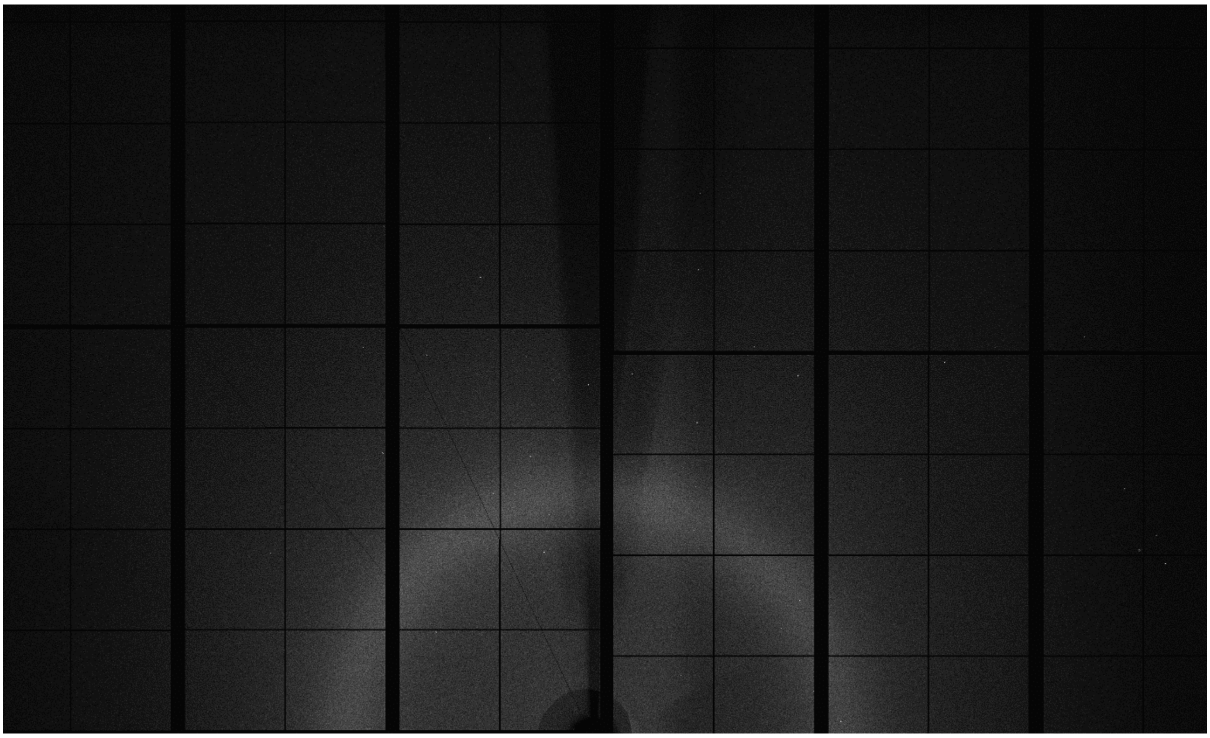

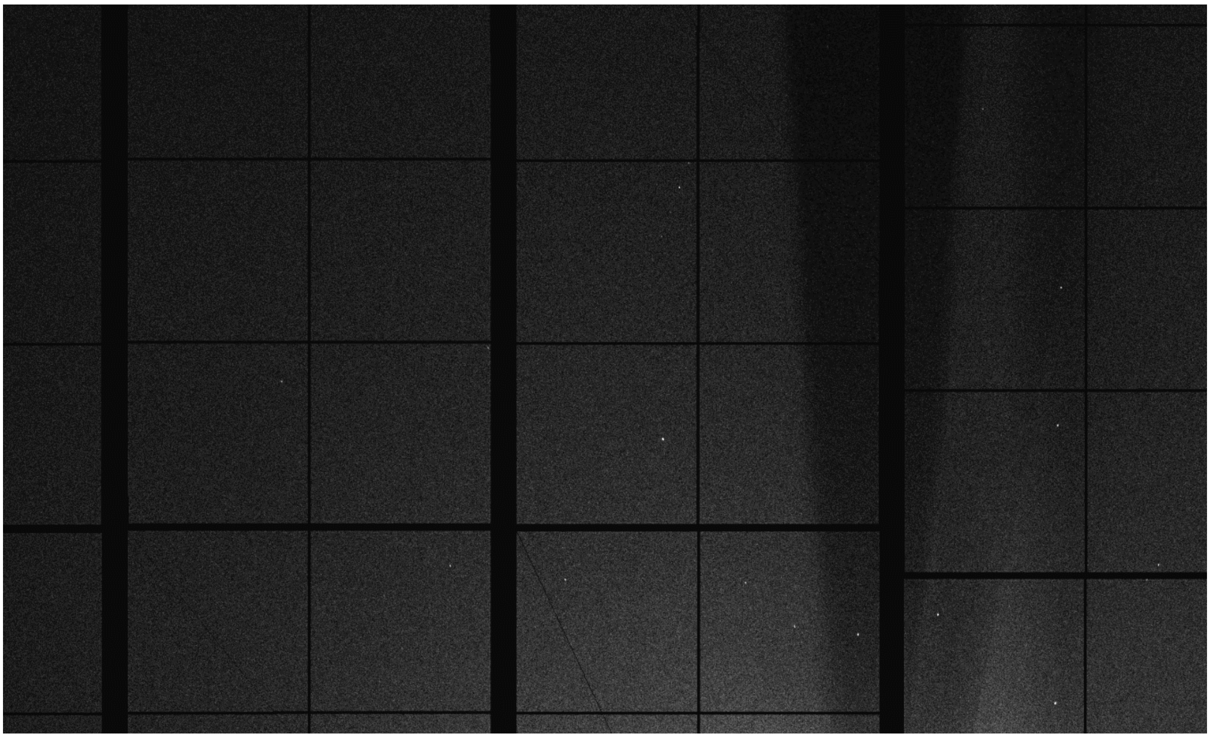

Supplement: Supplementary file 1 [file m-08-00905-sup1.pdf]
